# Synthesis of recyclable polyesters via a *cis*-fused ring strategy

**DOI:** 10.1093/nsr/nwaf516

**Published:** 2025-11-18

**Authors:** Ruiheng Gao, Guoquan Liu, Zhaoming Zhang, Xuzhou Yan, Shan Tang, Bin Wang

**Affiliations:** State Key Laboratory of Synergistic Chem-Bio Synthesis, Frontiers Science Center for Transformative Molecules, Shanghai Key Laboratory for Molecular Engineering of Chiral Drugs, School of Chemistry and Chemical Engineering, Shanghai Jiao Tong University, Shanghai 200240, China; State Key Laboratory of Synergistic Chem-Bio Synthesis, Frontiers Science Center for Transformative Molecules, Shanghai Key Laboratory for Molecular Engineering of Chiral Drugs, School of Chemistry and Chemical Engineering, Shanghai Jiao Tong University, Shanghai 200240, China; State Key Laboratory of Synergistic Chem-Bio Synthesis, Frontiers Science Center for Transformative Molecules, Shanghai Key Laboratory for Molecular Engineering of Chiral Drugs, School of Chemistry and Chemical Engineering, Shanghai Jiao Tong University, Shanghai 200240, China; State Key Laboratory of Synergistic Chem-Bio Synthesis, Frontiers Science Center for Transformative Molecules, Shanghai Key Laboratory for Molecular Engineering of Chiral Drugs, School of Chemistry and Chemical Engineering, Shanghai Jiao Tong University, Shanghai 200240, China; State Key Laboratory of Synergistic Chem-Bio Synthesis, Frontiers Science Center for Transformative Molecules, Shanghai Key Laboratory for Molecular Engineering of Chiral Drugs, School of Chemistry and Chemical Engineering, Shanghai Jiao Tong University, Shanghai 200240, China; Tianjin Key Laboratory of Composite & Functional Materials, School of Materials Science and Engineering, Tianjin University, and State Key Laboratory of High-Performance Roll Materials and Composite Forming, Tianjin 300350, China

**Keywords:** *cis*-fused lactones, chemical recyclability, ring strain engineering, cyclobutane rings, polyester

## Abstract

The development of chemically recyclable polymers is critical for addressing the environmental and resource limitations of the traditional linear plastic economy. Owing to its intrinsic reversibility, ring-opening polymerization (ROP) offers great potential for the development of recyclable polymers. However, achieving a balance between monomer polymerizability, polymer performance and recyclability remains a key challenge. This study explores the design and synthesis of a novel *cis*-fused cyclobutane-butyrolactone monomer (C4GBL) for use in ROP, leading to the formation of a polymer with 1,2-cyclobutane units in the main chain. C4GBL was synthesized using ethylene and bio-based maleic anhydride, and the resultant polymer exhibits high thermal stability (*T*_d_,_5%_ = 380°C). Thermodynamic analysis of the ROP of C4GBL revealed a ceiling temperature (*T*_c_) of −24°C, indicating that both polymerization and chemical recycling are feasible under appropriately tuned conditions. Density functional theory optimization reveals that the rigid fused cyclobutane forces the lactone segment of C4GBL into a near-eclipsed conformation, enhancing ring strain and polymerization reactivity. Collectively, this work establishes the *cis*-fused ring strategy as a new design principle for recyclable polyesters.

## Teaser

This study reports a strategy to synthesize recyclable polyesters using cis-fused rings, whose unique structure allows polymerization under mild conditions and efficient depolymerization through reversible isomerization.

## INTRODUCTION

The widespread production and consumption of plastics under a traditional linear economic model have resulted in severe environmental consequences and the depletion of finite resources [1–4]. To mitigate these challenges, closed-loop chemical recycling has emerged as a promising strategy, wherein polymers can be efficiently depolymerized into their original monomers or oligomers and subsequently repolymerized into materials with properties indistinguishable from their virgin counterparts [5–7]. This approach enables the retention of material performance over multiple use cycles and supports the transition toward a circular plastics economy. Ring-opening polymerization (ROP), due to its inherent reversibility, has emerged as a powerful platform for enabling such chemically recyclable systems [8,9]. Recent advances have demonstrated that precise control over polymerization thermodynamics—specifically the ceiling temperatures (*T*_c_)—can enable efficient ‘monomer ↔ polymer’ closed-loop systems [10–15]. Despite these advances, a central challenge persists: balancing monomer reactivity and polymer stability to optimize both recyclability and material performance [16]. Addressing this challenge at scale further requires the design of monomers that are not only tailored for closed-loop recycling but are also inexpensive, synthetically accessible and preferably derived from non-food biomass. The integration of thermodynamic recyclability with sustainable monomer sourcing is thus essential to realize truly practical and scalable circular polymer systems.

**Scheme 1.**
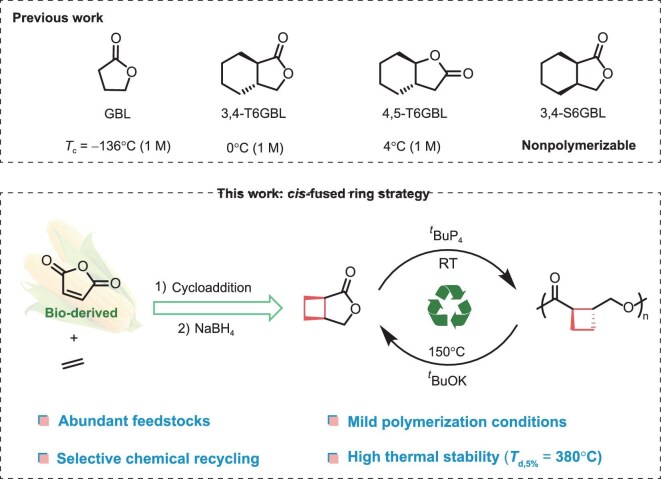
Ring-fused lactone monomers for ROP.

Among potential candidates, five-membered *γ*-butyrolactone (GBL) has attracted attention due to its derivation from renewable feedstocks and its ability to undergo ROP to yield poly(*γ*-butyrolactone) [P(GBL)]. Notably, P(GBL) is chemically equivalent to poly(4-hydroxybutyrate) [P(4HB)], a biopolymer traditionally produced via microbial fermentation. A synthetic route to P(GBL) thus avoids the drawbacks of fermentation, including poor reproducibility, low yields and high costs [17]. However, GBL has a very low *T*_c_ of −136°C (Scheme 1), such that polymerization must be conducted at 10 mol L^−1^ and −40°C, thereby severely limiting its practical applicability [18–21]. Accordingly, two approaches have been developed to address the performance limitations and render the five-membered lactone ring more polymerizable while preserving the intrinsic recyclability of the GBL core, including ring-fusing and atom-bridging [22–25]. In particular, the ring-fusion strategy has shown promise in tuning monomer thermodynamics to enable efficient ROP without compromising recyclability.

Haba and co-workers first demonstrated that *trans*-cyclohexyl-fused GBL (T6GBL) derivatives could undergo ROP under strong basic conditions, albeit with low polymer molar masses and poor polymerization control [26]. Furthermore, Zhu, Chen and colleagues systematically investigated *trans*-fused six-membered GBL analogs (Scheme 1), revealing that such a strategy significantly raises *T*_c_ values (e.g. *T*_c_ = 0°C for 3,4-T6GBL and 4°C for 4,5-T6GBL), enabling controlled polymerization under mild conditions. However, their corresponding *cis*-fused counterparts, such as 3,4-S6GBL, showed no polymerizability under either basic or coordination–insertion conditions [27,28]. Despite the clear success of *trans*-fused systems, the fundamental reasons why *cis*-fused analogs fail to polymerize—and how one might overcome those limitations—remain unaddressed.

Herein, we report the design and synthesis of a novel *cis*-fused cyclobutene-butyrolactone (C4GBL) monomer (Scheme 1) derived from readily available ethylene and bio-based maleic anhydride. C4GBL undergoes efficient polymerization under mild conditions catalyzed by the superbase *^t^*BuP_4_. In the presence of an external initiator, the process affords linear polyesters, whereas initiator-free conditions favor the formation of cyclic polymers. The resulting polyester features 1,2-cyclobutane units incorporated into the main chain and exhibits remarkable thermal stability (*T*_d,5%_ = 380°C). Notably, the polymer can be thermally depolymerized in the bulk state using *^t^*BuOK, regenerating the monomer in 82% isolated yield. To our knowledge, this represents the first example of a chemically recyclable polymer system based on a *cis*-fused GBL scaffold. Furthermore, density functional theory (DFT) calculations elucidate the origin of the enhanced ring strain in C4GBL and establish key structure-polymerizability correlations within this class of monomers. Collectively, these results highlight C4GBL as a versatile and scalable platform for developing high-performance, sustainable and chemically recyclable polymers.

## RESULTS AND DISCUSSION

Ethylene and maleic anhydride have been reported to efficiently undergo a [2 + 2] photocycloaddition reaction to form *cis*-3-oxabicyclo[3.2.0]heptane-2,4-dione [29–32], which can be reduced by sodium borohydride to produce the cyclic lactone (1*R*,5*S*)-3-oxabicyclo[3.2.0]heptan-2-one (C4GBL, Scheme 1). Initial polymerization of C4GBL was carried out using tris[*N,N*-bis(trimethylsilyl)amide]lanthanum [La(N(SiMe_3_)_2_)_3_] as the catalyst—known to promote the polymerization of GBL and its derivatives with benzyl alcohol (BnOH) as the initiator [18,27], but it was unsuccessful at both room temperature and at −40°C. Subsequent attempts used alternative catalytic systems, including zinc-based catalysts, ZnPh_2_/DBU and 1,5,7-triazabicyclodec-5-ene (TBD); however, these trials similarly failed to initiate polymerization (Table S1).

After evaluating several highly active catalysts for the ROP of C4GBL, an organocatalyst with extremely high basicity, 1-*tert*-butyl-4,4,4-tris(dimethylamino)-2,2-bis[tris(dimethylamino)-phosphoranylidenamino]-2λ^5^,4λ^5^-catenadi(phosphazene) (*^t^*BuP_4_) was tested for the anionic ROP of C4GBL. We initiated our investigation by conducting the ROP of C4GBL in tetrahydrofuran (THF) using diphenylmethanol (Ph_2_CHOH, p*K*a = 13.5) as the initiator. Under the condition of [M]/[Ph_2_CHOH]/[*^t^*BuP_4_] = 200:1:1, a polymer with number-average molecular weight (*M*_n_) of 13.8 kDa and a dispersity (*M*_w_/*M*_n_) of 1.48 (Table 1, entry 1) was obtained. With the goal of enhancing polymerization control, we systematically investigated the impact of solvents. The low-polarity solvent toluene yielded similar monomer conversion but a reduced molecular weight of 9.4 kDa, with *M*_w_/*M*_n_ increasing to 1.61 (Table 1, entry 2). In the highly polar solvent dimethylformamide (DMF), polymerization activity was significantly diminished, with only 19% monomer conversion (Table 1, entry 3). This observation can be ascribed to the strong solvation of DMF, which stabilizes both the cationic and anionic species and consequently lowers the nucleophilicity of the active anions, leading to a suppressed propagation reaction. Bulk polymerization at room temperature afforded 82% monomer conversion and a higher molecular weight of 19.1 kDa; however, a broader molecular weight distribution was observed (*M*_w_/*M*_n_ = 1.67) (Table 1, entry 4). Based on these findings, THF was selected as the solvent for subsequent studies. To investigate the influence of initiator acidity on polymerization behavior, we examined two weakly acidic initiators: BnOH (p*K*a = 15.4) and *tert*-butyl alcohol (*^t^*BuOH, p*K*a = 16.0) [33]. Under identical polymerization conditions, both initiators enabled high monomer conversions. However, a slight decrease in the *M*_n_ was observed as the initiator acidity decreased—10.7 kDa for BnOH and 9.3 kDa for *^t^*BuOH (Table 1, entries 5 and 6).

**Table 1.** Results of ROP of C4GBL under various conditions.^a^

Entry^a^	Catalyst	Initiator	Solvent	Time (h)	Conversion^b^ (%)	*M*_n,theo._^c^ (kDa)	*M*_n,SEC_^d^ (kDa)	*M*_w_/*M*_n_^d^	*cis*/*trans*
1	*^t^* BuP_4_	Ph_2_CHOH	THF	0.5	70	15.7	13.8	1.48	5/95
2	*^t^* BuP_4_	Ph_2_CHOH	TOLUENE	0.5	69	15.5	9.4	1.61	4/96
3	*^t^* BuP_4_	Ph_2_CHOH	DMF	0.5	19	4.3	n. d.	n. d.	
4	*^t^* BuP_4_	Ph_2_CHOH		0.5	82	18.4	19.1	1.67	5/95
5	*^t^* BuP_4_	BnOH	THF	0.5	66	14.8	10.7	1.44	5/95
6	*^t^* BuP_4_	*^t^* BuOH	THF	0.5	65	14.6	9.3	1.46	4/96
7	*^t^* BuP_4_		THF	1.0	69		19.3	1.54	5/95
8	*^t^* BuP_1_		THF	48					
9	*^t^* BuP_2_		THF	48	29		1.9	1.31	5/95
10	CH_3_ONa		THF	48					
11	*^t^* BuOK		THF	1.0	67		14.2	1.53	5/95
12	KHMDS		THF	1.0	68		8.7	1.52	5/95
13^e^	*^t^* BuP_4_		THF	2.0	81		24.2	1.55	4/96

a Conditions: [M]:catalyst:initiator = 200:1:1; [M] = 10 mol L^−1^ in solvent (0.3 g, 2.68 mmol); 17°C. The base and initiator (if present) were premixed prior to the addition of C4GBL. ^b^Determined by ^1^H NMR spectroscopy. ^c^Calculated from [C4GBL]_0_/[Initiator]_0_ × Conversion × *M*_w,C4GBL_ + *M*_w,I_. theo, theoretical. ^d^Determined by size exclusion chromatography (SEC) using polystyrene standards for calibration. ^e^Conditions: [M]:catalyst = 200:1; [M] = 10 mol L^−1^ in THF (2.0 g, 17.8 mmol); −10°C.

**Figure 1.**
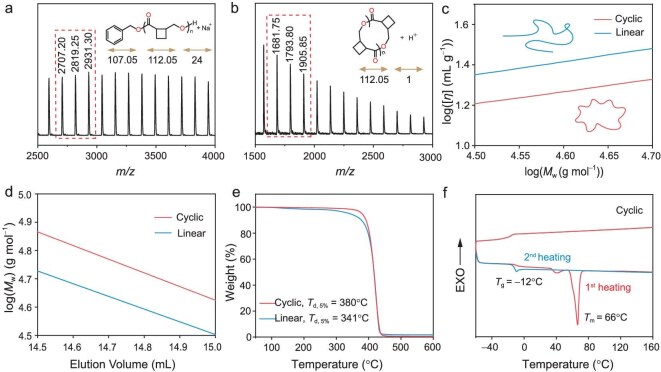
(a) MALDI-TOF-MS spectrum of P(C4GBL) produced by ([M]/[BnOH]/[*^t^*BuP_4_] = 200:1:1). (b) MALDI-TOF-MS spectrum of P(C4GBL) produced by ([M]/[*^t^*BuP_4_] = 200/1). (c) Double logarithm (Mark–Houwink–Sakurada) plots of intrinsic viscosity [*η*] versus absolute *M*_w_ of linear PC4GBL (*M*_n_ = 10.7 kDa, *M*_w_/*M*_n_ = 1.44) and cyclic P(C4GBL) (*M*_n_ = 19.3 kDa, *M*_w_/*M*_n_ = 1.54). (d) Logarithmic plots of *M*_w_ versus the elution volume of linear P(C4GBL) (*M*_n_ = 10.7 kDa, *M*_w_/*M*_n_ = 1.44) and cyclic P(C4GBL) (*M*_n_ = 19.3 kDa, *M*_w_/*M*_n_ = 1.5). (e) TGA curves of linear and cyclic P(C4GBL). (f) DSC curves of cyclic P(C4GBL).

The structure of the obtained polymer was characterized by ^1^H NMR, ^13^C NMR and matrix-assisted laser desorption/ionization time-of-flight mass spectrometry (MALDI-TOF MS). For example, the ^1^H NMR spectrum of P(C4GBL) confirmed a linear structure with high end-group fidelity (Fig. S5). This was further supported by the MALDI-TOF MS spectrum, which displayed a single series of molecular ion peaks with a uniform mass interval corresponding to the exact molar mass of the C4GBL (112.05 Da) repeat unit and featuring BnO/H as the end groups (Fig. 1a). The ^13^C NMR spectrum of P(C4GBL) in the carbonyl region displayed a major resonance at 174.2 ppm and a minor resonance at 173.4 ppm (Fig. S6), corresponding to two distinct carbonyl environments [34]. Subsequent heteronuclear single quantum coherence (HSQC) and heteronuclear multiple bond correlation (HMBC) analyses confirmed that the minor ^1^H resonances correlate directly with the minor ^13^C resonance, with chemical shifts closely matching those of the major set (Figs S7 and S8). These observations indicate that the major–minor pattern arises from *cis–trans* isomerization. To verify whether this pattern originated from monomer isomerization before polymerization, an *in situ* NMR experiment was performed by mixing the C4GBL with *^t^*BuP_4_ (Fig. S9). The ^1^H NMR spectrum of C4GBL remained unchanged after 1 h, indicating that the *cis* monomer does not isomerize to the *trans* form before polymerization. Therefore, we propose that the observed major–minor pattern arises from *cis–trans* isomerization occurring after polymerization, consistent with literature reports of *^t^*BuP_4_-induced epimerization in ROP [35–37]. Mechanistically, under the strongly basic conditions provided by *^t^*BuP_4_, *α*-protons adjacent to the carbonyl are deprotonated to a carbanion intermediate (Scheme S1). This intermediate permits partial rotation about the C–C bond, enabling interconversion between *cis* and *trans* configurations, with the *trans* isomer thermodynamically favored (Fig. S10).

Notably, polymerization proceeded efficiently in the absence of an external initiator with *^t^*BuP_4_ as the sole catalyst (Table 1, entry 7), affording P(C4GBL) with *M*_n_ = 19.3 kDa and *M*_w_/*M*_n_ = 1.54. A central question was whether the polymerization proceeded via *α*-deprotonation of the lactone carbonyl—similar to GBL—to yield linear polymers [20], or if cyclic polymers were preferentially formed under these conditions. Polymer topology was first examined by ^1^H NMR spectroscopy (Fig. S11), which showed no signal attributable to terminal hydroxymethylene protons, indicating the absence of chain-end functionalities. This observation was further corroborated by MALDI-TOF MS (Fig. 1b), which exhibited a single series of molecular ion peaks with a uniform mass spacing of 112.05 Da—corresponding precisely to the repeat unit of C4GBL—and no detectable mass contribution from end groups, consistent with a cyclic architecture. To further verify and characterize the linear and cyclic topologies, both forms of the polymer were analyzed by gel permeation chromatography (GPC) equipped with triple detection, including light scattering, refractive index, and viscometry detectors. The resultant Mark–Houwink–Sakurada double-logarithmic plot, portraying the correlation between intrinsic viscosity ([*η*]) and absolute weight-average molecular weight (*M*_w_) for P(C4GBL) of both topologies, indicates that the cyclic polymers displayed a lower intrinsic viscosity at the same *M*_w_, and the [*η*]_cyclic_/[*η*]_linear_ ratio was found to be 0.7, consistent with the theoretically predicted value and the experimentally observed value for other cyclic polymer (Fig. 1c) [10,38,39]. In addition, the logarithm plots of *M*_w_ versus the elution volume reveal that the cyclic P(C4GBL) has smaller hydrodynamic volume (Fig. 1d). A plausible ring-closing mechanism is proposed in Scheme S2. Although *^t^*BuP_4_ exhibits weak nucleophilicity, it can initiate the anionic polymerization of C4GBL to produce linear polymers at the beginning. The subsequent protic quenching step induces intramolecular ring-closing along with displacement of the terminal *^t^*BuP_4_ group, ultimately yielding the cyclic polymer [10]. Kinetic studies further indicate that the polymerization is well-controlled during the initial 30 min, as evidenced by the linear increase of *M*_n_ with monomer conversion (Fig. S13). As the reaction proceeds, the increasing occurrence of intramolecular cyclization reduces the uniformity of propagation, leading to a gradual loss of control.

Building on the above results, we expanded our investigation to a broader range of organic bases to catalyze ROP of C4GBL and to evaluate their ability to produce cyclic polymer topologies. Accordingly, several representative strong bases, including *^t^*BuP_1_, *^t^*BuP_2_, CH_3_ONa, *^t^*BuOK and potassium hexamethyldisilazide (KHMDS), were examined. When *^t^*BuP_1_ and CH_3_ONa were used, no monomer conversion was observed (Table 1, entries 8 and 10), indicating that their basicity is insufficient to initiate the polymerization. Polymerization with *^t^*BuP_2_ proceeded slowly, resulting in only 29% monomer conversion after 48 h and affording a polymer with an *M*_n_ of 1.9 kDa (Table 1, entry 9). In contrast, *^t^*BuOK and KHMDS catalyzed significant monomer conversion (67% and 68%, respectively) within 1 h. MALDI-TOF MS analyses (Figs S14 and S15) of the purified polymers confirmed the formation of cyclic topologies in both cases, although the resulting polymers exhibited relatively low *M*_n_ values of 14.2 and 8.7 kDa, respectively (Table 1, entries 11 and 12). Furthermore, in separate larger-scale experiments at −10°C, the monomer conversion reached 81%, affording a polymer with an *M*_n_ of 24.2 kDa (entry 13), slightly exceeding the theoretical value. The observed differences in molecular weight can be attributed to the distinct catalytic profiles of the bases. Compared to *^t^*BuOK and KHMDS, *^t^*BuP_4_ more efficiently promotes monomer activation due to its higher basicity. In parallel, its exceptional bulk (∼1.4 nm) suppresses intramolecular backbiting, delaying cyclization and enabling the formation of cyclic polymers with significantly higher molar masses [10,40]. Notably, cyclic polymers exhibit slightly higher *M*_n_ than their linear counterparts at the same monomer conversion, which can be explained by the relatively low initiation efficiency of *^t^*BuP_4_ under initiator-free conditions, leading to fewer polymer chains and consequently longer chains.

The thermal stability of P(C4GBL) was assessed using thermogravimetric analysis (TGA), which revealed exceptional stability under a nitrogen atmosphere. The cyclic polymer exhibited a 5% weight loss temperature (*T*_d,5%_) as high as 380°C, indicating outstanding thermal resistance (Fig. 1e). In comparison, the linear polymer showed a slightly lower *T*_d,5%_ of 341°C, but still demonstrated good thermal performance. Notably, the thermal stability of P(C4GBL) is comparable to that of a recently reported polyester bearing 1,3-cyclobutyl units in its backbone and surpasses that of all previously reported circular aliphatic or aromatic polyesters [41,42]. These results highlight the crucial role of cyclobutyl incorporation in enhancing the thermal stability of aliphatic polyesters. The thermal properties of P(C4GBL) were further investigated using differential scanning calorimetry (DSC). The linear and cyclic polymers exhibited comparable DSC profiles, indicating similar thermal transitions and overall structural features. The glass transition temperature (*T*_g_) of the polymers synthesized by both methods was consistently measured at −12°C (Fig. 1f). Notably, the polymer exhibited a pronounced melting transition with a peak temperature of 66°C in the first heating scan. However, no melting temperature (*T*_m_) was observed in the second heating cycle, even when the experiment was conducted at a reduced heating/cooling rate of 5°C min^−1^ (Fig. S17). This absence of *T*_m_ can be attributed to the low degree of isotacticity, which leads to a slow crystallization rate. The semi-crystalline nature of P(C4GBL) was further supported by wide-angle X-ray scattering (WAXS) analysis, which revealed three distinct groups of diffraction peaks at 2*θ* = 15°, 19° and 21°. The degree of crystallinity was determined to be 44.2% (Fig. S18).

To gain deeper insight into the polymerization mechanism, the thermodynamics of C4GBL ROP were systematically investigated by quantifying the equilibrium monomer concentration ([M]_eq_) at various temperatures using *^t^*BuP_4_ as the catalyst. (Table S2). This analysis yielded standard-state thermodynamic data for polymerization with an enthalpy change (Δ*H*°_p_) of −17.0 kJ mol^−1^ and an entropy change (Δ*S*°_p_) of −68.1 J mol^−1^ K^−1^ (Fig. S19), as derived from the equation ln[M]_eq_ = Δ*H*°_p_/*RT* − Δ*S*°_p_/*R*. The *T*_c_ was determined to be −24°C at [M]_0_ = 1.0 mol L^−1^, based on Dainton’s equation (*T*_c_ = Δ*H*_p_/(Δ*S*_p_ + *R*ln[M]_0_), where *R* is the gas constant). Although the monomer and polymer adopt different configurations (Figs S20–S27), the determination of *T*_c_ is based on the enthalpy (Δ*H*) and entropy (Δ*S*) of polymerization and is independent of the polymer configuration [43]. Therefore, the measured equilibrium conversions and derived thermodynamic parameters accurately reflect the monomer–polymer equilibrium, and the resulting *T*_c_ is considered valid. As summarized in Table 2, the absolute value for Δ*H*_p_ of C4GBL is higher than that of GBL (Δ*H*_p_ = −5.4 kJ mol^−1^) [18], and is in closer proximity to the values observed for *trans*-fused ring monomers, such as 4,5-T6GBL and 3,4-T6GBL (Δ*H*_p_ = −18 and −20 kJ mol^−1^, respectively) [27,28]. These findings suggest that the increased ring strain energies (RSEs) in C4GBL relative to those in GBL contribute to its enhanced polymerizability, while its thermodynamic characteristics more closely resemble those of *trans*-fused monomer systems.

**Table 2.**
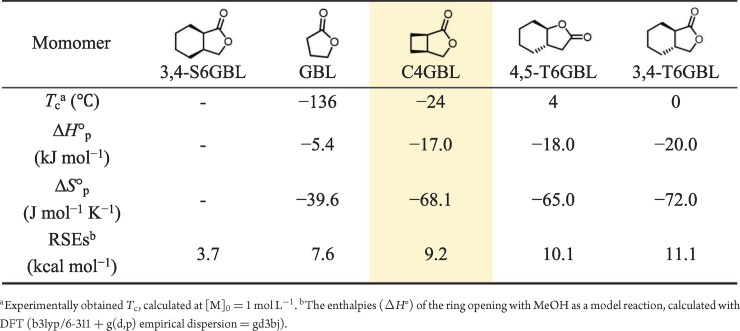
Thermodynamic parameters and ring strain for the ROP of GBL and its derivatives.

To further understand the effect of ring fusion on the ring strain of GBL-based scaffolds, DFT calculations were performed using methanol as a model nucleophile. *Cis*-cyclohexane-fused GBL (3,4-S6GBL), which is known to be a non-polymerizable monomer, exhibits a low ring strain of 3.7 kcal mol^−1^. In contrast, C4GBL shows a significantly higher ring strain of 9.2 kcal mol^−1^, which is slightly lower than those of the *trans*-fused analogues. These results are consistent with the experimental observations, indicating that increased ring strain correlates with enhanced polymerizability. Overall, these thermodynamic insights demonstrate that incorporating a fused *cis*-cyclobutane ring effectively increases the ring strain of lactone monomers. Thus, in addition to the conventional strategy of introducing *trans*-fused rings, *cis*-cyclobutane fusion offers a promising alternative for tuning monomer reactivity in the design of chemically recyclable polymer.

**Figure 2.**
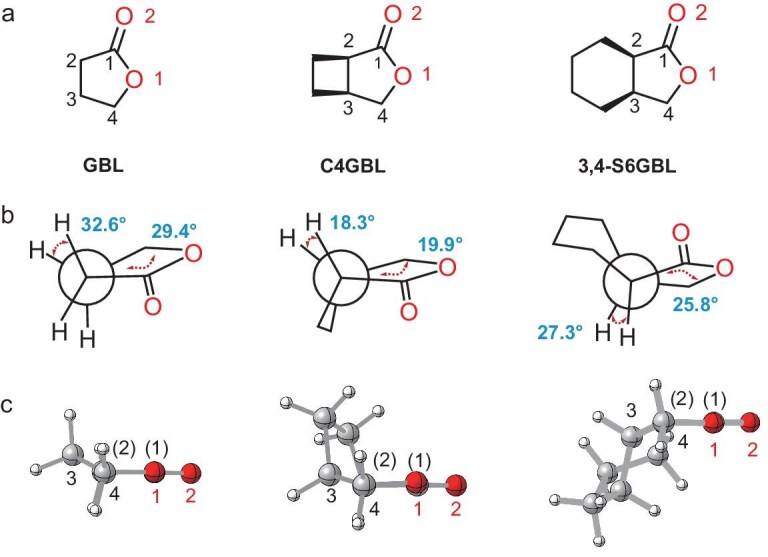
(a) Structures of GBL, C4GBL and 3,4-S6GBL with labeled carbon and oxygen atoms. (b) The Newman projections for GBL, C4GBL and 3,4-S6GBL are shown, with the dihedral angles H–C_2_–C_3_–H and C_2_–C_3_–C_4_–O_1_ indicated alongside each projection. (c) Optimized conformations of GBL, C4GBL and 3,4-S6GBL.

The influence of ring fusion on the RSEs of the GBL motif was investigated by comparing the optimized geometries of GBL with those of four fused-ring derivatives. Owing to their intrinsic RSEs, five-membered lactones preferentially adopt non-planar envelope or twist conformations. In the two *trans*-fused derivatives (3,4-T6GBL and 4,5-T6GBL), distortion of the cyclohexane ring enforces twist conformations in the lactone ring, which are inherently less stable (Fig. S35). In contrast, GBL and the *cis*-fused analogues adopt more favorable envelope conformations. This conformational disparity contributes to the increased ring strain observed in the *trans*-fused systems. Comparison of geometrical parameters for GBL and the two *cis*-fused lactones shows that their bond lengths and angles are largely comparable, implying that RSE differences arise mainly from torsional strain (dihedral distortions) [44]. In C4GBL, the dihedral angles between H–C_2_–C_3_–H bonds in the five-membered ring (18.3°) and between the C_1_–C_2_–C_3_–C_4_ bonds (16.8°), as well as between the C_2_–C_3_–C_4_–O_1_ bonds (19.9°), are markedly smaller than the corresponding values in GBL (32.6°, 27.3°, 29.4°) and 3,4-S6GBL (27.3°, 23.7°, 25.8°) (Fig. 2b). These significantly smaller dihedral angles indicate that C4GBL adopts an overall conformation closer to an eclipsed geometry, resulting in enhanced torsional strain [45]. The elevated ring strain in C4GBL originates from its rigid cyclobutane-fused structure, which restricts conformational flexibility and forces multiple dihedral angles into near-eclipsed conformations, making it difficult to relieve strain effectively. In contrast, six-membered rings possess high conformational flexibility and can adopt chair-like or distorted conformations to alleviate localized strain. As a result, even when certain dihedral angles approach eclipsed geometries, other parts of the molecule can adjust to redistribute and partially offset the strain, leading to an overall lower ring strain compared to GBL.

**Figure 3.**
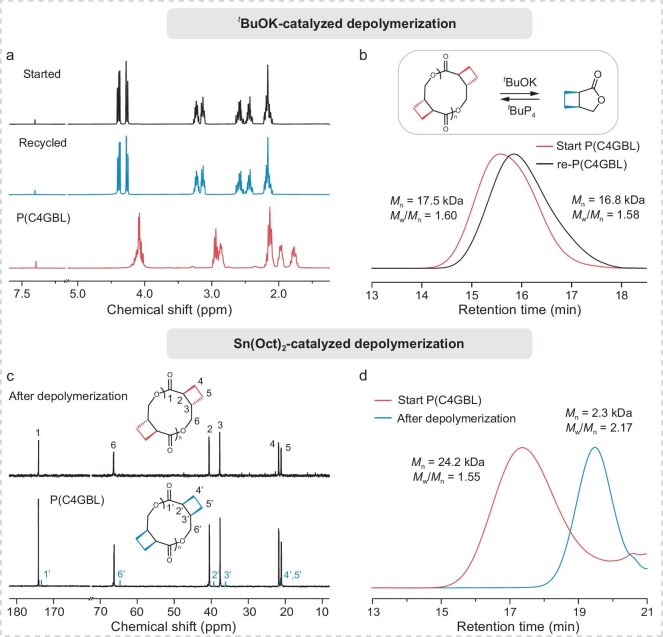
(a) Overlay of the ^1^H NMR spectra in CDCl_3_ of P(C4GBL) (bottom), recycled monomer after depolymerization (middle) and starting monomer as a reference (top). (b) SEC traces of the initial P(C4GBL) and recycled P(C4GBL) (eluent, THF; flow rate, 1.0 mL min^−1^). (c) Overlay of the ^13^C NMR spectra (CDCl_3_) of P(C4GBL) (bottom, containing both *cis* repeat units, highlighted in blue, and *trans* repeat units) and the depolymerization residue obtained from Sn(Oct)_2_ catalyzed degradation (top, pure *trans* configuration), with the reaction performed at 150°C using 5 mol% Sn(Oct)_2_. (d) SEC traces of the initial P(C4GBL) and the Sn(Oct)_2_-catalyzed depolymerization residue (eluent, THF; flow rate, 1.0 mL min^−1^).

As previously mentioned, a *T*_c_ of −24°C was calculated for [M]₀ = 1 mol L^−1^ using Dainton’s equation. This relatively low *T*_c_ suggests the potential for easy chemical recycling of the polyesters. To explore this, the bulk depolymerization of P(C4GBL) synthesized by ROP with an *M*_n_ of 17.5 kg mol^−1^ was investigated for monomer recovery. Given the monomer–polymer equilibrium inherent to this system, we hypothesized that a strong organic base would be required to promote epimerization during depolymerization and enable efficient recovery of C4GBL from P(C4GBL). Heating the polymer at 150°C under reduced pressure in the presence of 5 wt% *^t^*BuOK catalyzed its depolymerization to the C4GBL monomer. The monomer was efficiently recovered in 82% yield by vacuum distillation, as confirmed by ^1^H NMR (Fig. S28). Trace impurities in the crude recovered monomer likely originate from partially *trans*-configured monomer units that did not undergo epimerization. These *trans* monomers may undergo slight decomposition or minor side reactions under the depolymerization and distillation conditions. After minimal purification (Fig. 3a), the recycled monomer could be readily repolymerized to afford P(C4GBL) with a molar mass comparable to that of the pristine polymer (Fig. 3b and Fig. S30). In contrast, depolymerization with Sn(Oct)_2_—which effectively suppresses epimerization during the process [46]—afforded only a small amount of C4GBL, and no ^1^H (Fig. S29) or ^13^C NMR (Fig. 3c) signals corresponding to *cis*-P(C4GBL) were detected in the depolymerized residue, further confirming that P(C4GBL) predominantly adopts the *trans* configuration. Moreover, it was shown that efficient monomer recovery requires *trans*-configured units to first epimerize to the *cis* configuration, as illustrated in Scheme S3, which compares the proposed depolymerization pathways under different catalytic conditions. GPC analysis of the depolymerized residue revealed an *M*_n_ of 2.3 kDa (Fig. 3d), indicating that *cis*-configured repeat units are randomly distributed along the polymer backbone. These experiments demonstrate that *cis–trans* isomerization has a significant impact on depolymerization efficiency and provides a mechanistic basis for establishing a ‘monomer–polymer–monomer’ closed-loop recycling system for C4GBL.

## CONCLUSION

In conclusion, the *cis*-fused cyclobutane-butyrolactone monomer (C4GBL) was successfully synthesized and polymerized via ROP to give a polyester amenable to chemical recycling. The introduction of a fused *cis*-cyclobutane ring into the monomer structure increases the ring strain, which both enhances polymerization activity and improves the thermal stability of the resulting polymer (*T*_d,5%_ = 380°C). Thermodynamic analysis revealed a moderate *T*_c_ of −24°C, which facilitates efficient polymerization and chemical recycling. Bulk depolymerization experiments demonstrate that *cis–trans* isomerization governs the efficiency of P(C4GBL) depolymerization. When *^t^*BuOK is used as the catalyst, *trans*-configured units along the polymer chain first epimerize into the *cis* configuration, followed by depolymerization to regenerate the monomer with an 82% yield. These results highlight C4GBL as a promising candidate for the development of sustainable polyesters, offering a new strategy for creating recyclable polymeric materials that contribute to a circular plastics economy. This study sets the foundation for future advancements in the design and recycling of bio-based, chemically recyclable polyesters.

## Supplementary Material

nwaf516_Supplemental_File
