# Supplementary material for: Synthesis of recyclable polyesters via a cis-fused ring strategy
Source: Natl Sci Rev. 2025 Nov 18;12(12):nwaf516. doi: 10.1093/nsr/nwaf516 (PMC12715863; doi:10.1093/nsr/nwaf516)
Supplement: nwaf516_Supplemental_File [file nwaf516_supplemental_file.pdf]

Supplementary Information

for

Synthesis of Recyclable Polyesters via a *cis*-Fused Ring Strategy

Ruiheng Gao[1], Guoquan Liu[1], Zhaoming Zhang[1], Xuzhou Yan[1]*, Shan Tang[1]* and Bin Wang[2]*

[1]State Key Laboratory of Synergistic Chem-Bio Synthesis, Frontiers Science Center for Transformative Molecules, School of Chemistry and Chemical Engineering, Shanghai Jiao Tong University, Shanghai 200240, P. R. China
E-mail: xzyan@sjtu.edu.cn, tang.shan@sjtu.edu.cn

[2]Tianjin Key Laboratory of Composite & Functional Materials, School of Materials Science and Engineering, Tianjin University, Tianjin 300350, P. R. China. State Key Laboratory of High-Performance Roll Materials and Composite Forming, Tianjin 300350, P. R. China.
E-mail: binwang@tju.edu.cn

Materials and general methods

Materials

Unless otherwise stated, all chemicals are used without further purification. Tris[N,N-bis(trimethylsilyl)amide]lanthanum ([La(N(SiMe$_3$)$_2$)$_3$], 97%), diphenylzinc (Zn(C$_6$H$_5$)$_2$, 99%), 1,5,7-triazabicyclo[4.4.0]dec-5-ene (TBD, 98%), 1,8-diazabicyclo[5.4.0]-undec-7-ene (DBU, 99%), Stannous octoate (Sn(Oct)$_2$, 95%) were purchased from Aladdin. 1-*tert*-butyl-4,4,4-tris(dimethylamino)-2,2-bis[tris(dimethylamino)-phosphoranylidenamino]-2λ^5,4λ^5-catenadi(phosphazene) (^{t}Bu-P$_4$, ~0.8 M in hexane), 1-*tert*-butyl-2,2,4,4,4-pentakis(dimethylamino)-2λ5,4λ5-catenadi(phosphazene) (^{t}Bu-P$_2$, ~2.0 M in THF) and *tert*-Butylimino-tris(dimethylamino)phosphorane (^{t}Bu-P$_1$) , were purchased from Aldrich Chemical Co. Potassium tert-butoxide (^{t}BuOK, 98%), sodium methoxide (CH$_3$ONa, 95%), potassium bis(trimethylsilyl)amide (KHMDS, 1.0 mol L^{-1} in THF) were purchased from Energy Chemical. Diphenylmethanol was purchased from Aldrich Chemical Co, which was purified by dissolving in toluene over CaH$_2$, filtering after an overnight stir, and collecting by evaporation of the solvent. Benzyl alcohol (BnOH, 99%) and *tert*-butanol (^{t}BuOH) obtained from Alfa Aesar Co. were stirred with CaH$_2$ for 24 hours then distilled under reduced pressure. Sodium borohydride (98%) was purchased from Sinopharm Chemical Reagent Co., Ltd.

Methods

Nuclear magnetic resonance (NMR)

^{1}H, ^{13}C, HSQC and HMBC NMR spectra were recorded on a Bruker Avance III spectrometer (400 MHz for ^{1}H NMR and 101 MHz for ^{13}C NMR) at room temperature in chloroform-*d* (purchased form Innochem), and chemical shifts values were referenced to chloroform-*d* as internal standard at 7.26 ppm or TMS as internal standard at 0 ppm for ^{1}H NMR and against chloroform-*d* at 77.16 ppm for ^{13}C NMR.

Gel permeation chromatography (GPC)

Unless otherwise noted, all GPC data were obtained using an Agilent PL-GPC 50 system at 40 °C with a flow rate of 1.0 mL min^{-1} using THF as the eluent and calibrated with polystyrene (PS) standards. The columns included a 5 μm PL gel guard (50 × 7.5 mm), a PL gel mixed-B (300 × 7.5 mm), and a PL gel mixed-C (300 ×

7.5 mm). Samples were filtered through 0.22 μm PTFE filters prior to analysis. The specific sample shown in Figure 3d, as well as the samples corresponding to Entries 1, 5, and 13, were analyzed using an HLC-8320 GPC (TOSOH, Japan) with THF as the eluent and PS standards for calibration.

Matrix-assisted laser desorption/ionization time-of-flight mass spectra (MALDI-TOF MS)

MALDI-TOF MS of typical polymer samples were performed on a Bruker Autoflex III mass spectrometer in linear, positive ion mode, using *trans*-2-[3-(4-*tert*-butylphenyl)-2-methylprop-2-enylidene]propanedinitrile (DCTB) and 2,5-dihdyroxybenzoic acid (DHB) as matrix, and THF as solvent.

Thermo-gravimetric analysis (TGA)

TGA was conducted on a thermogravimetric analyzer (America TA TGA550) at a heating rate of 10 °C min^{-1} under a nitrogen flow of 100 mL min^{-1}. Decomposition onset temperature (T_d) was defined at 5% mass loss.

Differential scanning calorimetry (DSC)

DSC was performed on a TA Q2000 differential scanning calorimeter. The heating and cooling rates were set at 10 °C min^{-1}. Thermograms were recorded and analyzed with a TA Universal Analysis software. Data of the endothermic thermograms were recorded from the first scan to give information about the as-prepared polymers and glass transition temperature (T_g) was recorded from the second heating scan.

Wide-angle X-ray diffraction (WAXD)

WAXD measurements were performed on the SAXSFocus 3.0 system (GKINST, China). The setup consists of a multilayer focused Cu K_α X-ray source (MFOCUS, GKINST, China). The X-ray wavelength was 0.154 nm. Two dimensional (2D) images were recorded using an EIGER2 R 1M detector, which had a resolution of 1062 × 1028 pixels (the pixel size of 75 μm × 75 μm).

Mechanical tests

The mechanical properties of the polymers were measured using an Instron 3343 universal testing machine under standard tensile conditions. Dog bone shaped specimens were prepared by slow solvent evaporation and tested at a crosshead speed of 5 mm min^{-1} at ambient temperature until break. The measurement was repeated at least three times, and the reported values represent the average of the obtained data.

Synthesis of C4GBL

cis-3-Oxabicyclo[3.2.0]heptane-2,4-dione was synthesized based on the literature procedures.[1] The product (8.0 g, 63.4 mmol) was dissolved in THF (120 mL) and $NaBH_4$ (3.6 g, 95.2 mmol) was added while stirring under argon. The mixture was cooled to −78 °C then MeOH (15 mL) was added dropwise over l h. The reaction mixture was stirred at the same temperature for 2 h, and then 1 M HCl (50 mL) and 6 M HCl (15 mL) were subsequently added. The mixture was stirred at room temperature for 0.5 h, then extracted with dichloromethane (6 × 60 mL). Drying ($MgSO_4$) and evaporation left the crude product. Purification by chromatography on silica gel (CH_2Cl_2) gave the product as a colorless oil (5.6 g, 79%).

^{1}H NMR ($CDCl_3$, 400 MHz) δ 2.01–2.17 (m, 2H), 2.29–2.43 (m, 1H), 2.44–2.58 (m, 1H), 3.01–3.10 (m,1H), 3.11–3.20 (m,1H), 4.22 (dd, 1H, J = 1.5, 9.6), 4.31 (dd, 1H, J = 6.4, 9.6); ^{13}C NMR ($CDCl_3$, 101 MHz) δ 23.5, 25.4, 34.3, 38.1, 74.2, 181.0.

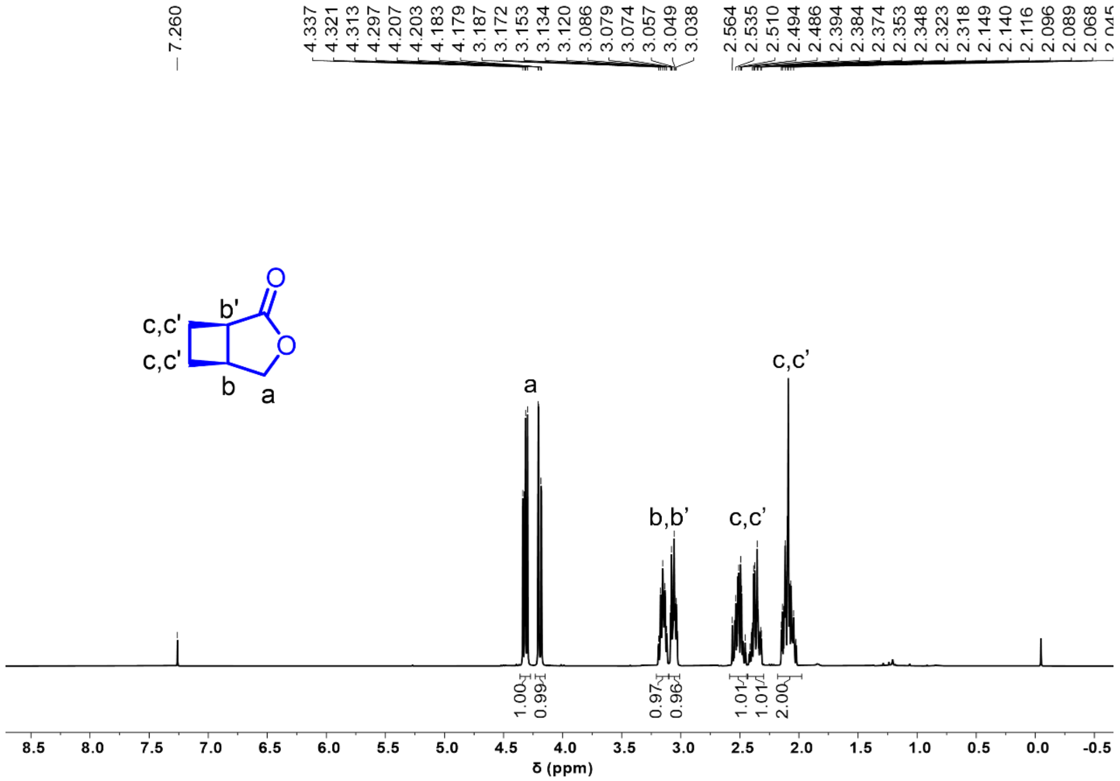

Figure S1. ^{1}H NMR (400 MHz, $CDCl_3$, 25 °C) spectrum of C4GBL.

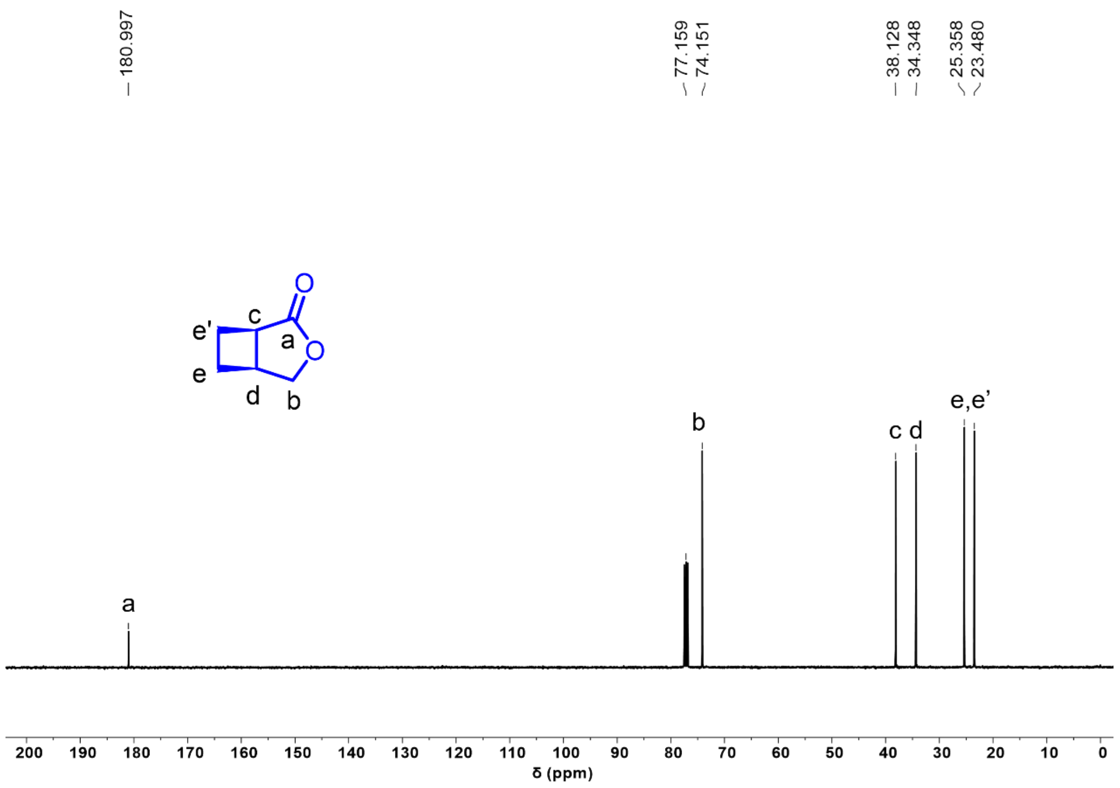

Figure S2. ^{13}C NMR (101 MHz, $CDCl_3$, 25 °C) spectrum of C4GBL.

Ring Opening Polymerization

Polymerization was carried out in a glovebox. A predetermined amount of initiator, solvent, and catalyst was added to the reactor and stirred for 10 minutes. The monomer C4GBL was then rapidly added to initiate the polymerization. After a predetermined reaction time, the polymerization was quenched by the addition of 0.5 mL of benzoic acid/chloroform solution (10 mg mL^{-1}). A 0.2 mL aliquot was taken from the reaction mixture for ^1H NMR analysis to determine the monomer conversion. The quenched reaction mixture was precipitated into 50 mL of cold methanol, filtered, and washed with methanol to remove unreacted monomer. The resulting polymers were dried under vacuum at 50 °C for 24 h. All analyses were performed on purified samples.

Table S1. Ring-opening polymerization of C4GBL

Entry[a]	*Cat.*	*Temp.* (°C)	Time (h)	*Conv.*[b] (%)	$M_{n,\ theo}$ (kDa)	$M_{n,\ SEC}$ (kDa)	M_w/M_n
1	$La[N(SiMe_3)_2]_3$	r.t.	12	-	-	-	-
2	$La[N(SiMe_3)_2]_3$	–40	12	-	-	-	-
3	Zn-1	r.t.	12	-	-	-	-
4	TBD	r.t.	12	-	-	-	-
5	DBU	r.t.	12	-	-	-	-
6	$DBU/ZnPh_2$	r.t.	12	-	-	-	-

[a] Conditions: [M]: *cat.*: BnOH = 100:1:1, [M] = 10 mol L^{-1} in THF (0.3 g, 2.7 mmol), base and initiator were mixed first, followed by monomer. [b] Determined by ^{1}H NMR spectroscopies.

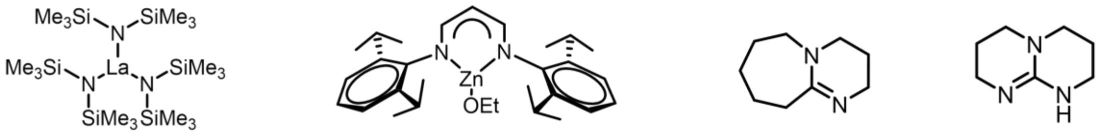

$La[N(SiMe_3)_2]_3$ Zn-1 DBU TBD

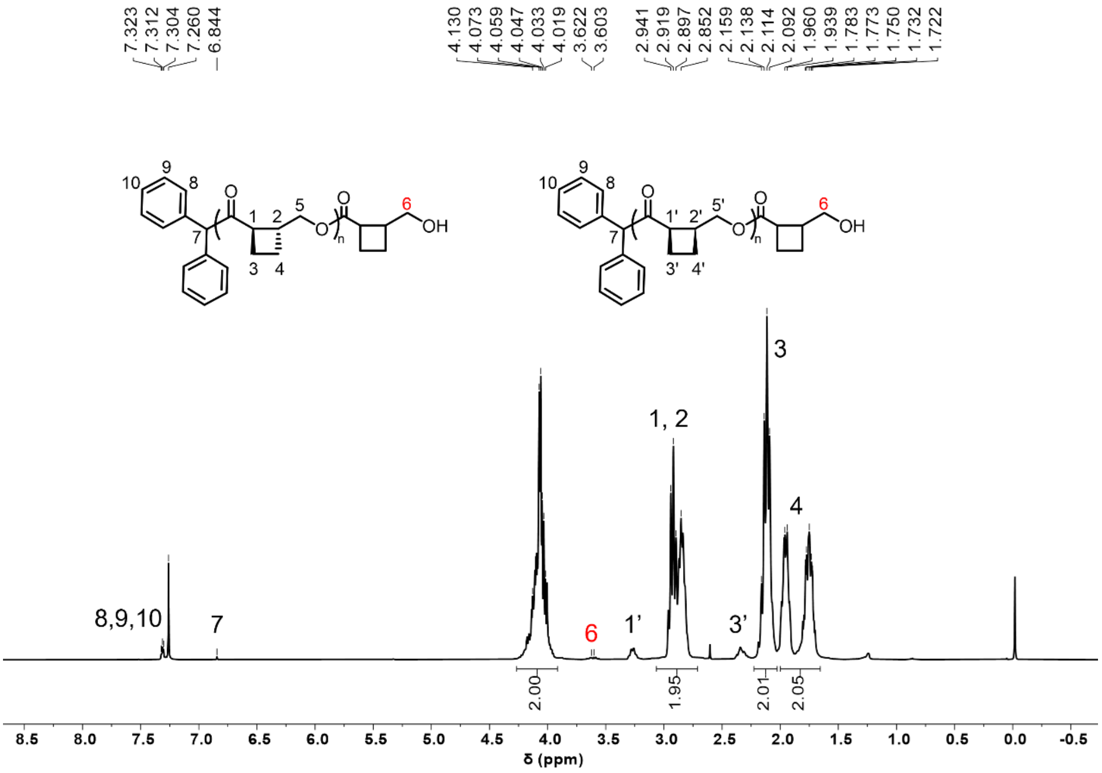

Figure S3. ^{1}H NMR (400 MHz, $CDCl_3$, 25 °C) spectrum of P(C4GBL) obtained by

mixing ^{t}Bu-P_4 and Ph_2CHOH first, then adding C4GBL (Entry 1, Table 1).

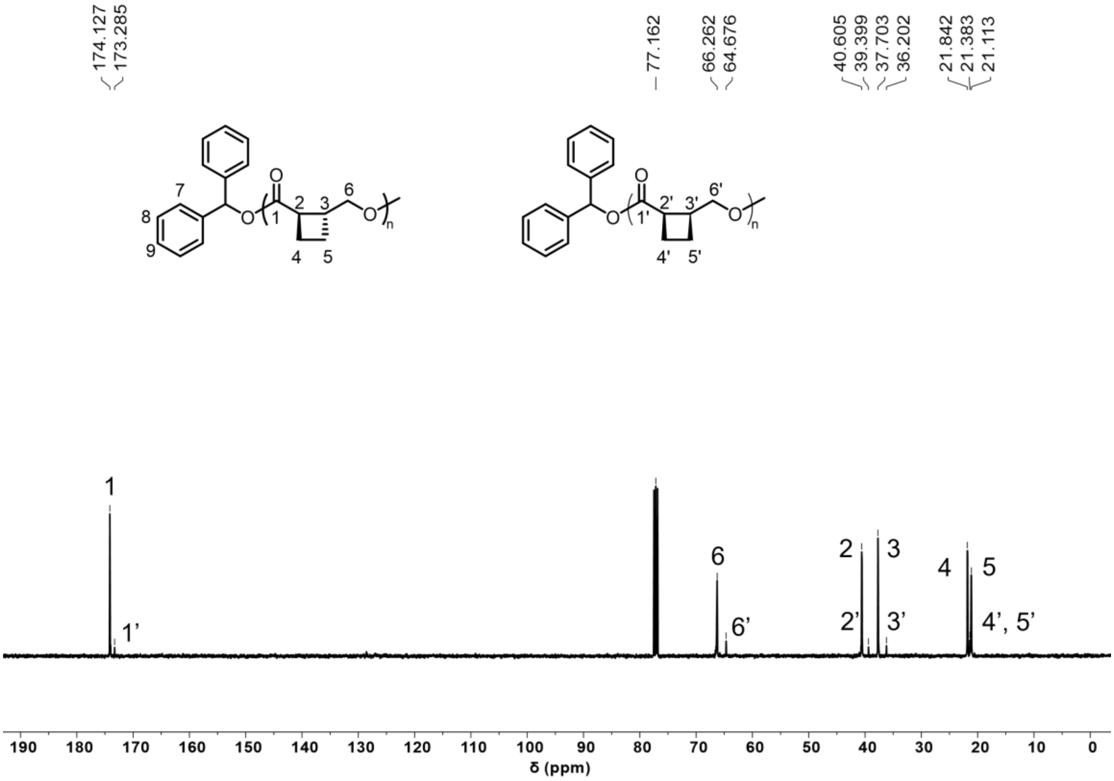

Figure S4. ^{13}C NMR (101 MHz, $CDCl_3$, 25 °C) spectrum of P(C4GBL) obtained by mixing ^{t}Bu-P_4 and Ph_2CHOH first, then adding C4GBL (Entry 1, Table 1).

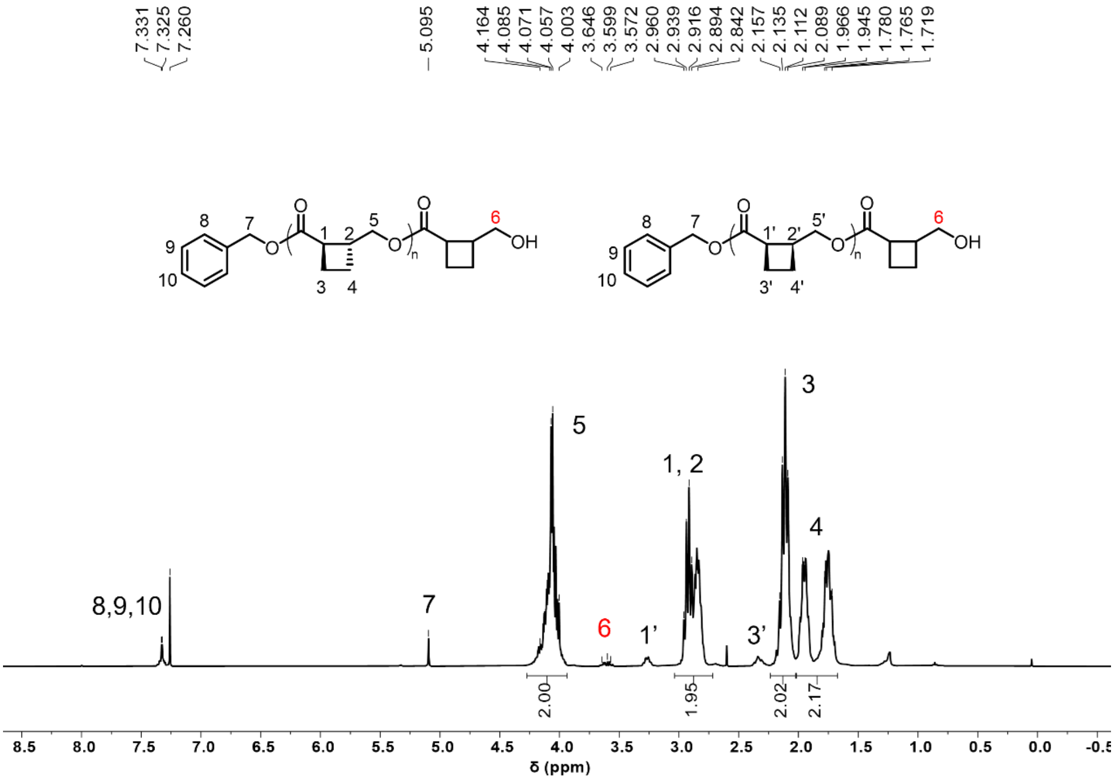

Figure S5. ^{1}H NMR (400 MHz, $CDCl_3$, 25 °C) spectrum of P(C4GBL) obtained by mixing ^{t}Bu-P_4 and BnOH first, then adding C4GBL (Entry 5, Table 1).

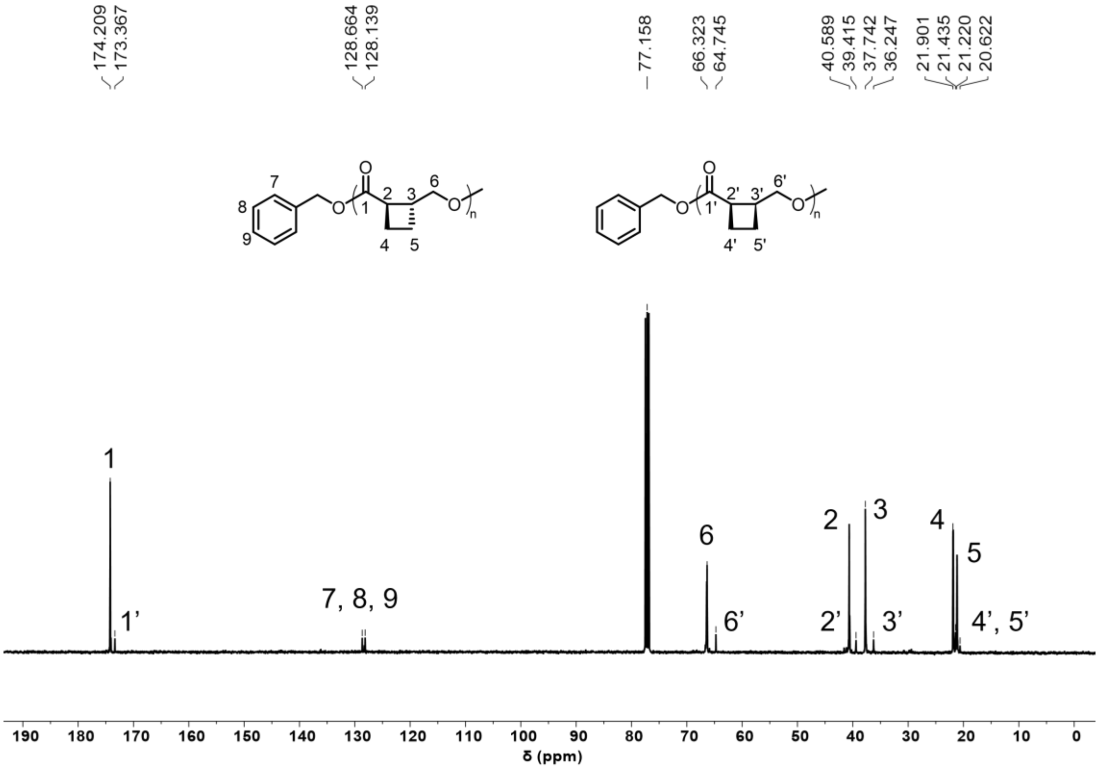

Figure S6. ^{13}C NMR (101 MHz, $CDCl_3$, 25 °C) spectrum of P(C4GBL) obtained by obtained by mixing ^{t}Bu-P_4 and BnOH first, then adding C4GBL (Table 1, Entry 5).

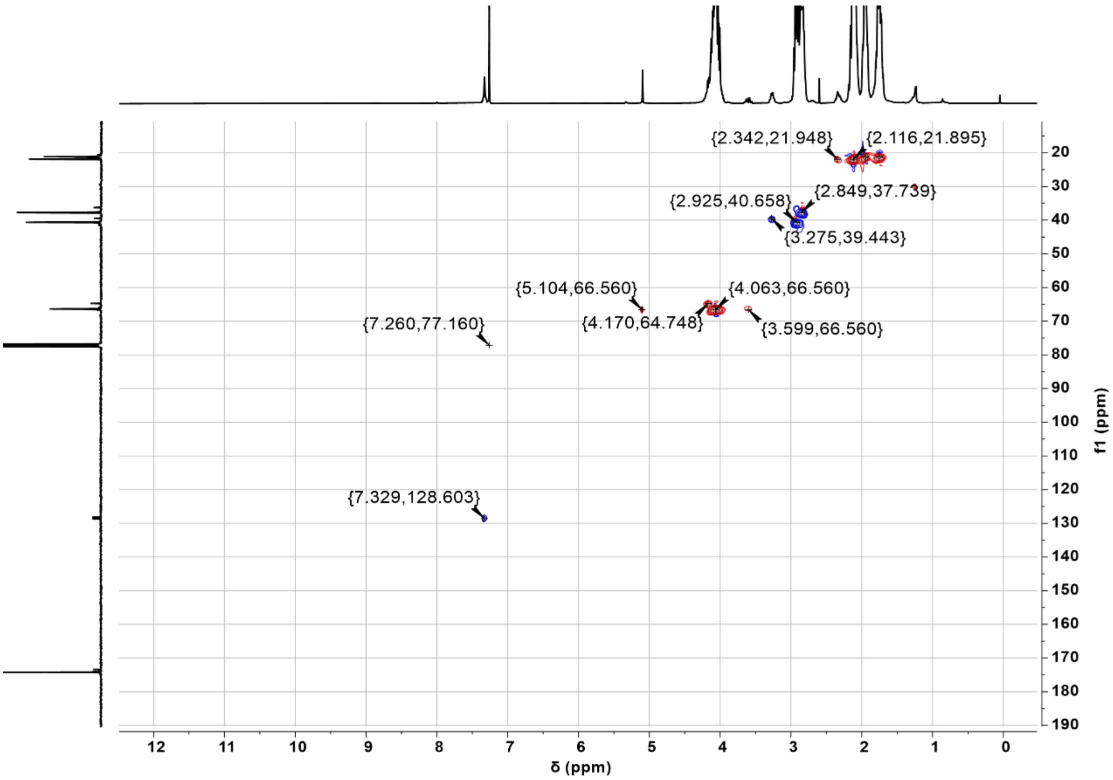

Figure S7. HSQC spectrum of P(C4GBL) obtained by obtained by mixing ^{t}Bu-P$_4$ and BnOH first, then adding C4GBL.

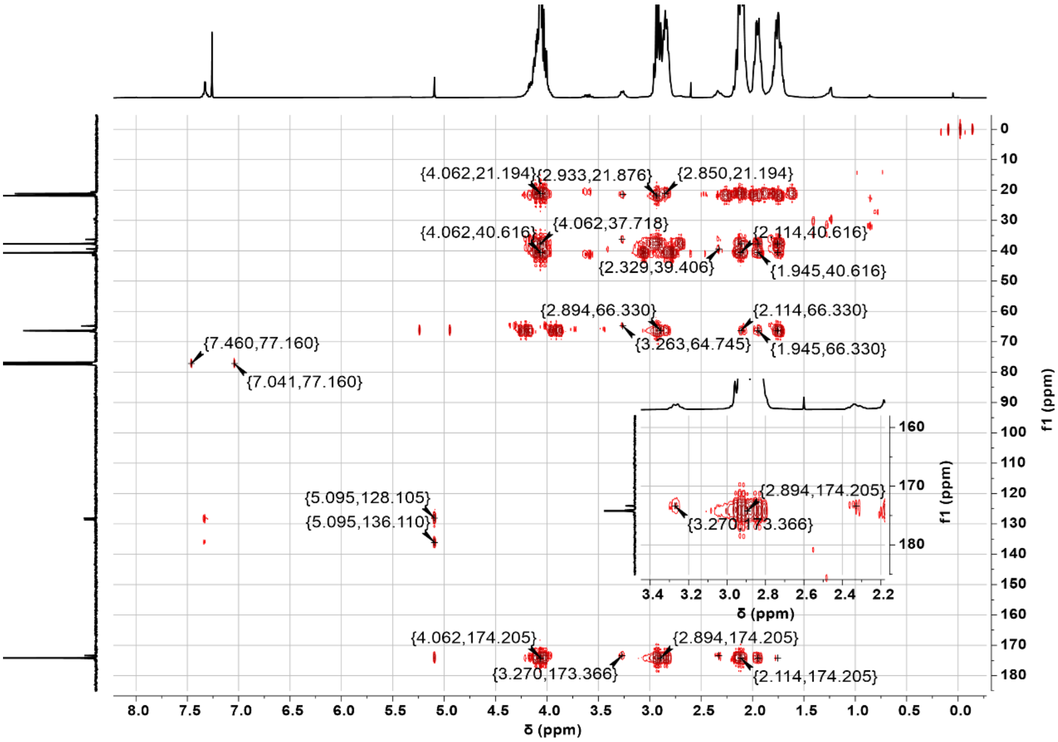

Figure S8. HMBC spectrum of P(C4GBL) obtained by obtained by mixing ^{t}Bu-P$_4$ and BnOH first, then adding C4GBL.

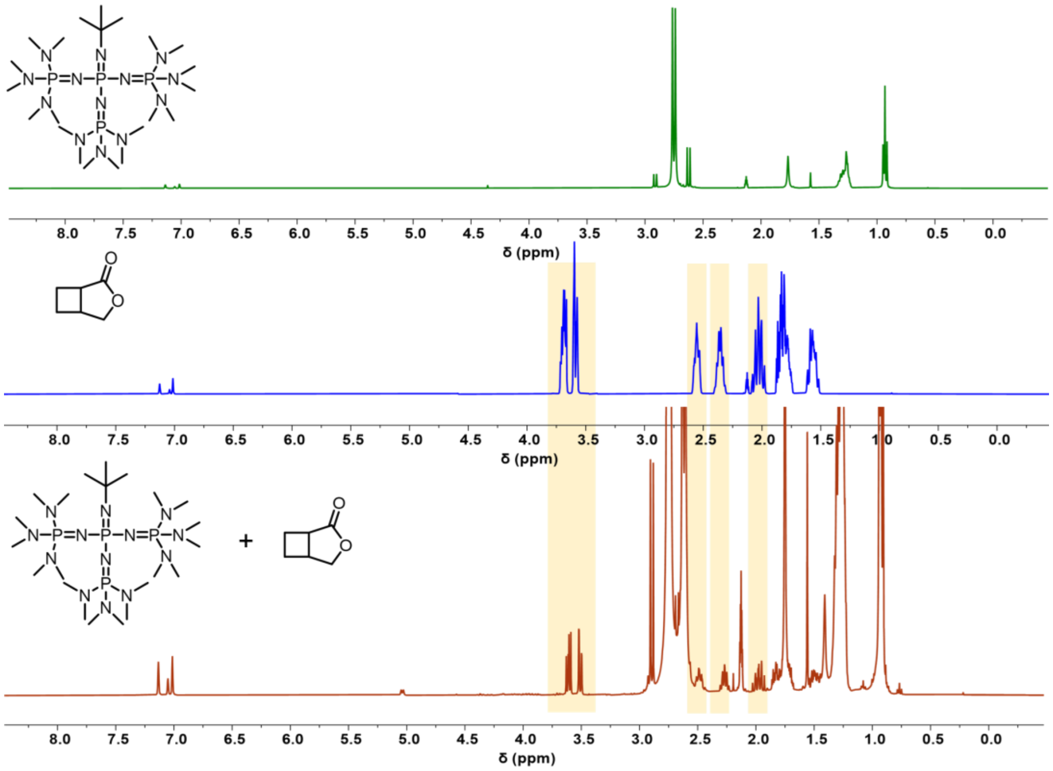

Figure S9. ^{1}H NMR (400 MHz, toluene-d_8, 25 ℃) spectrum of the reaction between ^{t}Bu-P_4 and C4GBL in a 1:1 ratio after 1h.

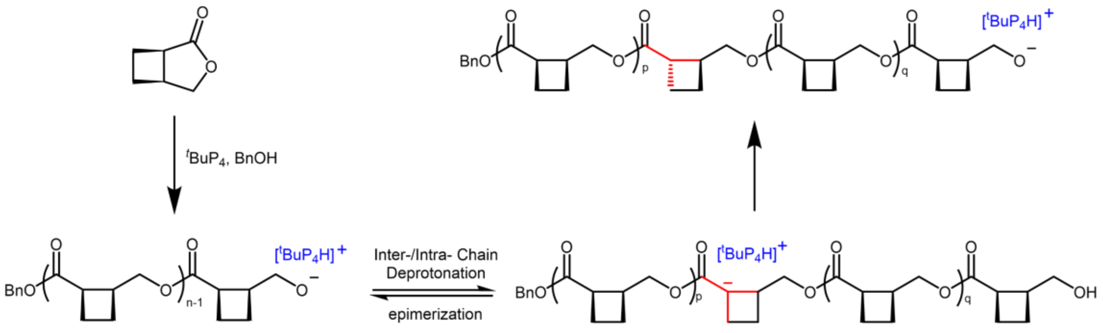

Scheme S1. Proposed mechanism of epimerization at the stereocenter adjacent to the carbonyl.

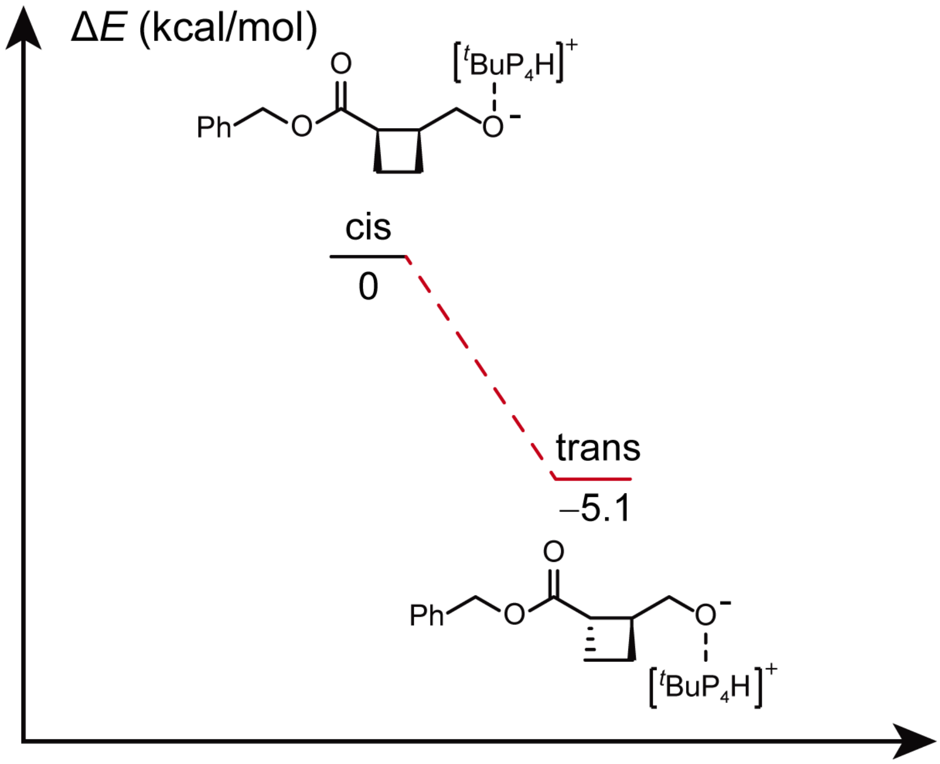

Figure S10. DFT-calculated energy profile of the *cis*-*trans* isomerization.

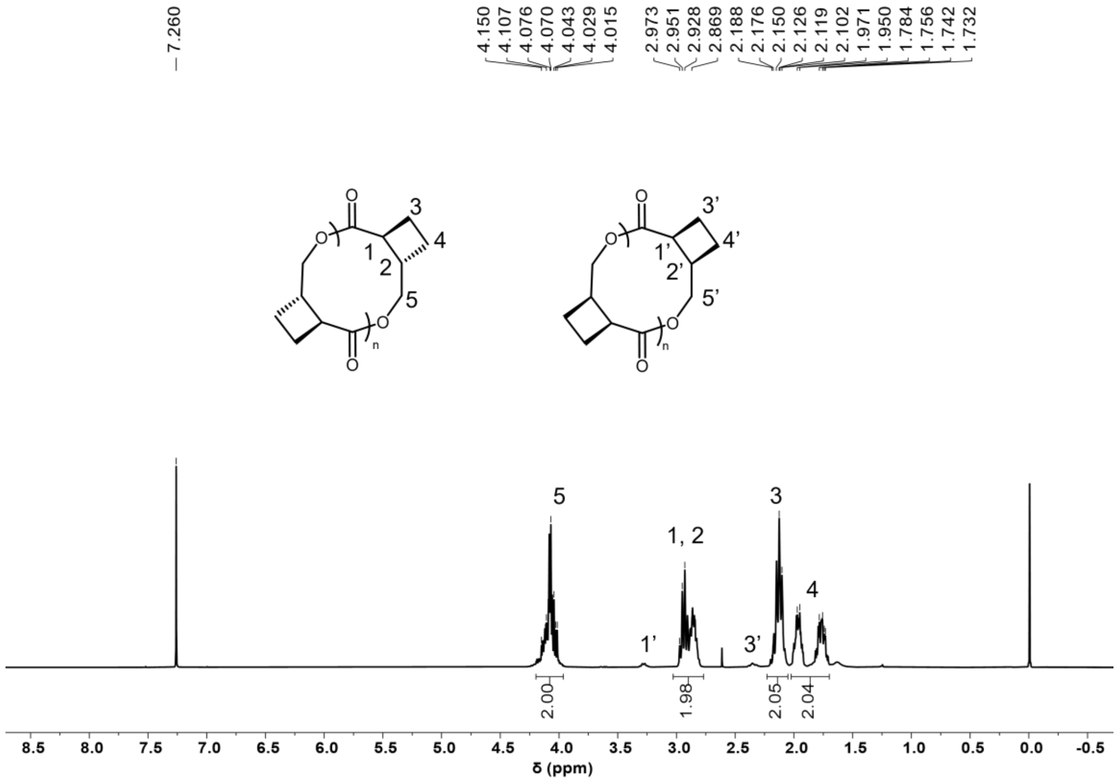

Figure S11. ^{1}H NMR (400 MHz, $CDCl_3$, 25 °C) spectrum of cyclic P(C4GBL) obtained by ^{t}Bu-P_4 alone (Table 1, Entry 7).

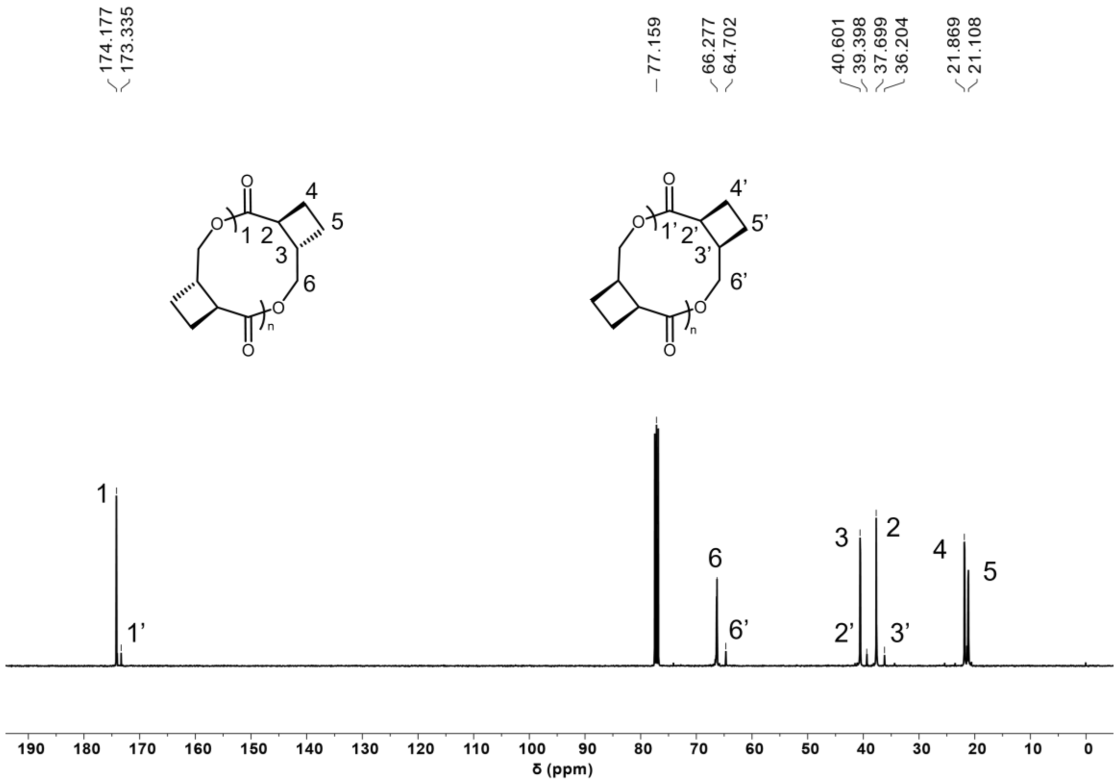

Figure S12. ^{13}C NMR (101 MHz, $CDCl_3$, 25 °C) spectrum of cyclic P(C4GBL) obtained by ^{t}Bu-P_4 alone (Table 1, Entry 7).

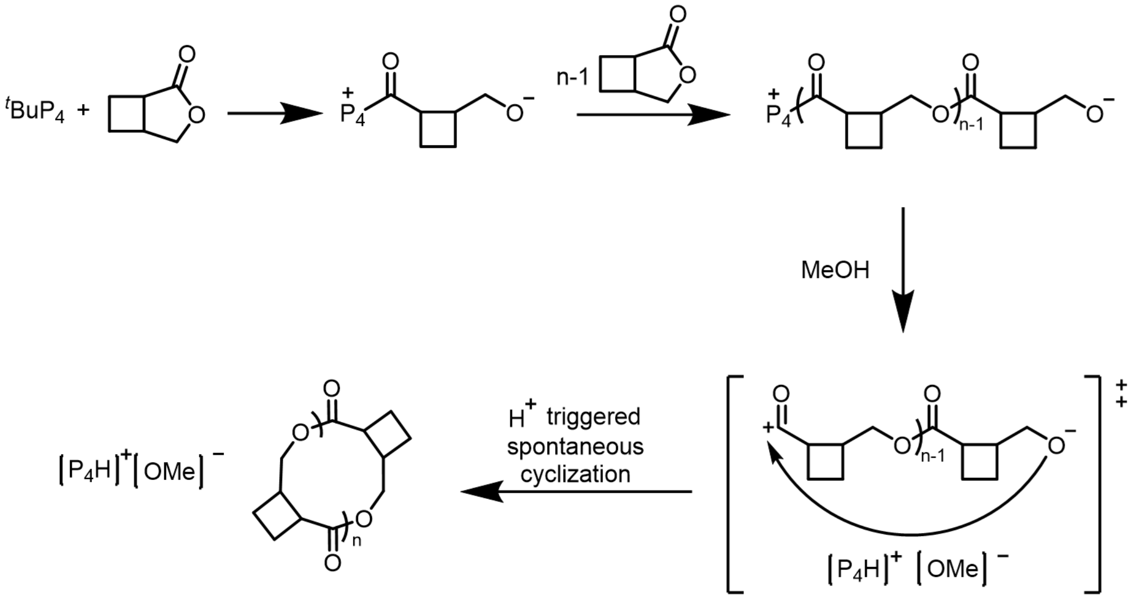

Scheme S2. Proposed mechanism for macromolecular cyclization triggered by a protic reagent MeOH. In-polymerization cyclization is sterically blocked by the bonded bulky superbase ($^{t}BuP_4$) moiety. However, the detachment of this bulky superbase on protonation allows cyclization to occur, producing c-P(C4GBL).

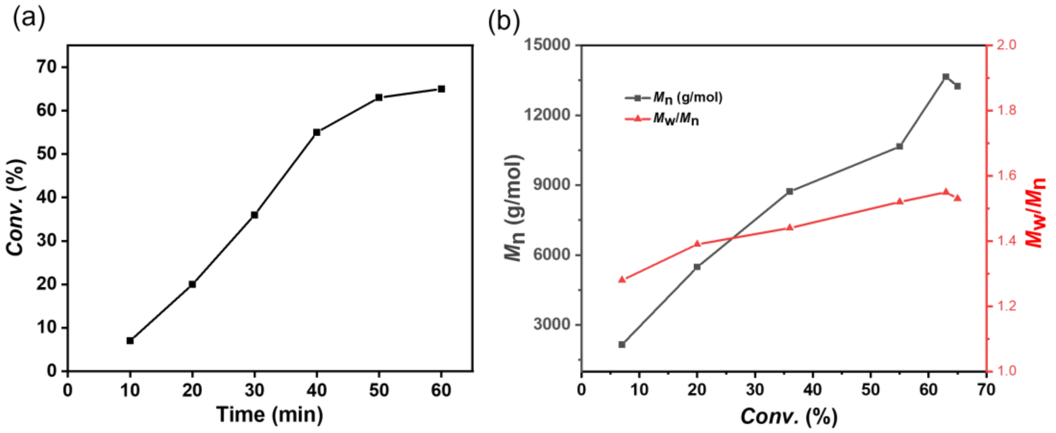

Figure S13. Kinetic plot for C4GBL polymerization: **a**, Dependence of conversion on polymerization time; **b**, dependence of molecular weight (M_n) and molecular weight distribution (M_w/M_n) on C4GBL conversion (conditions: C4GBL/^{t}Bu-P_4 = 200/1, THF, 24 °C, 10 mol L^{-1}).

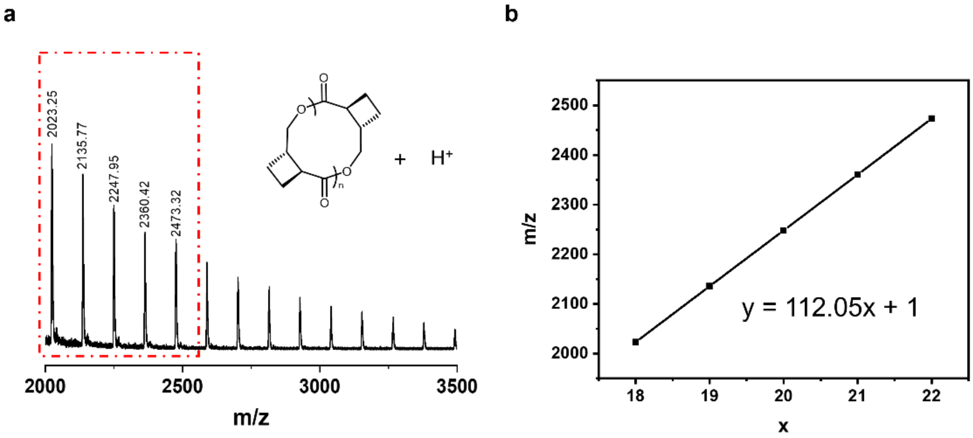

Figure S14. **a**, MALDI-TOF mass spectrum of PC4GBL produced directly by ^{t}BuOK. **b**, plots of *m*/*z* values *vs* the number of C4GBL repeat units (x). Note: MALDI-TOF typically measures lower molecular weights than GPC due to its limitations. It is more suited for analyzing low molecular weight species, and for higher molecular weight samples, weaker signal intensity and increased fragmentation lead to an underestimation of the molecular weight. Additionally, during ionization, high molecular weight components may fragment, further lowering the measured value.

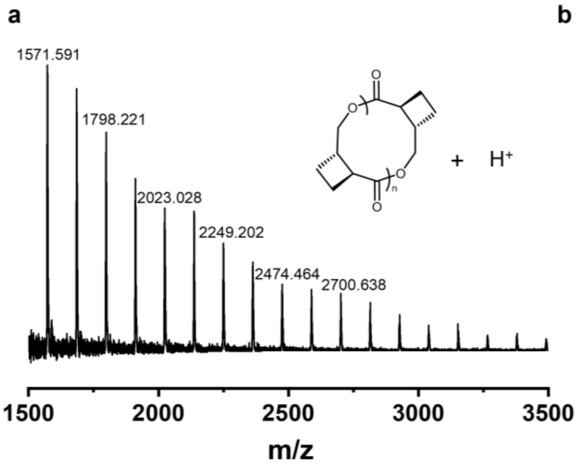

Figure S15. **a**, MALDI-TOF mass spectrum of PC4GBL produced directly by KHMDS and **b**, plots of *m*/*z* values *vs* the number of C4GBL repeat units (x).

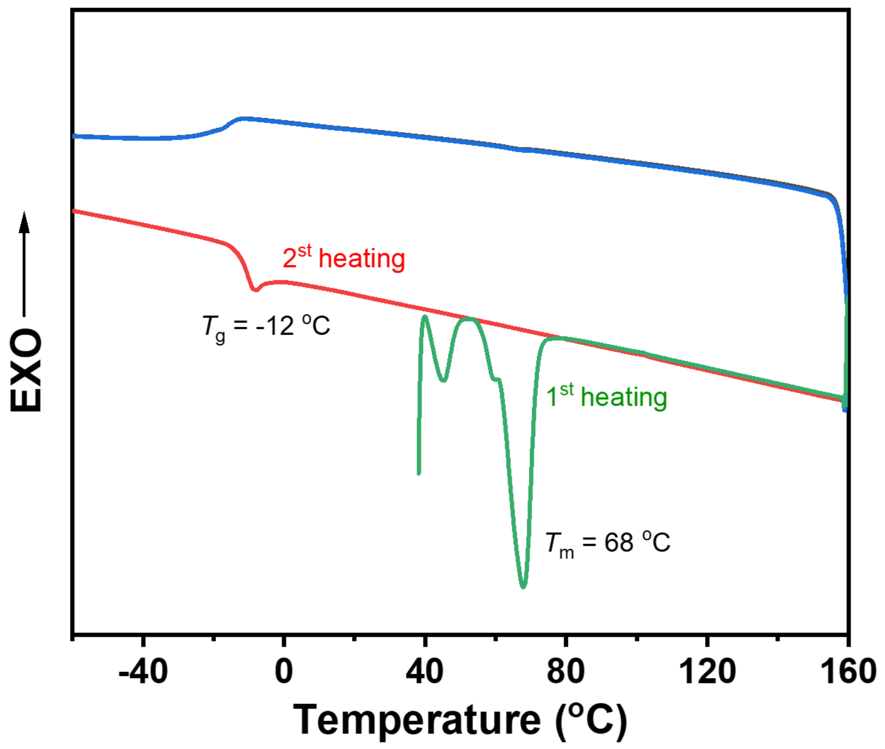

Figure S16. DSC curves of linear PC4GBL.

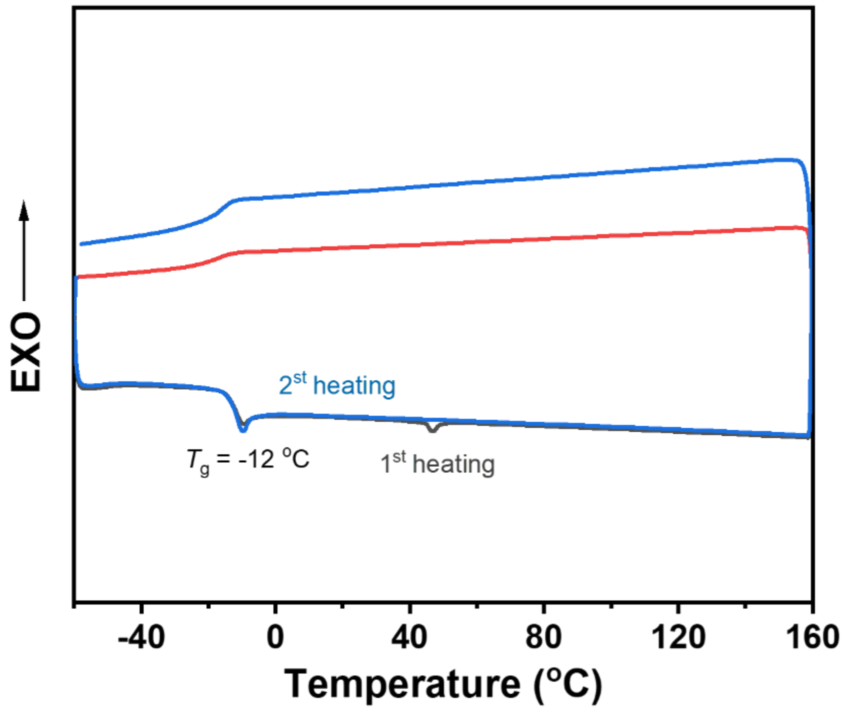

Figure S17. DSC curves of cyclic PC4GBL at a cooling rate of 5 °C min^{-1}.

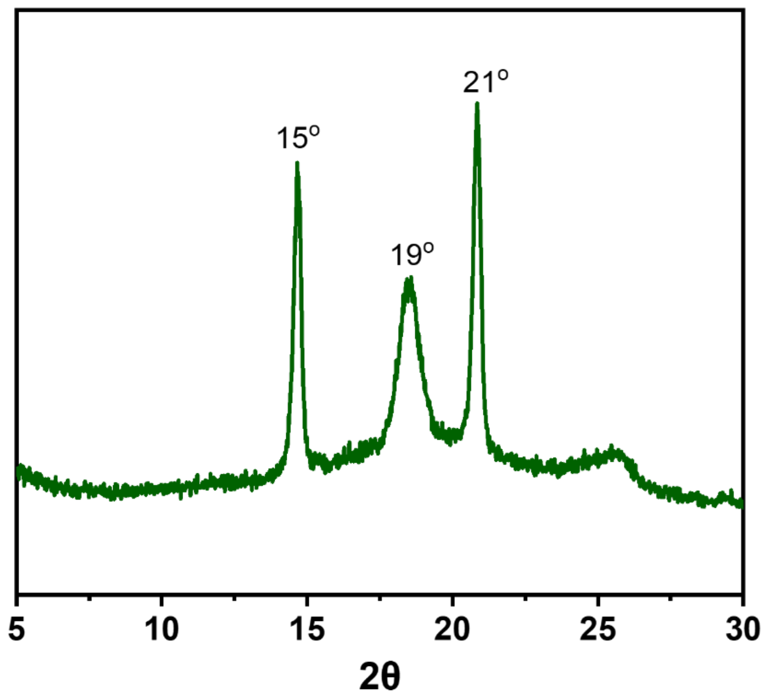

Figure S18. XRD curves of cyclic PC4GBL.

Thermodynamics of the ROP of C4GBL

Table S2. Equilibrium monomer concentrations measured at various temperatures.

Entry[a]	*Temp.*(°C)	*Temp.*(K)	Time (h)	*Conv.*[b] (%)	M_{eq}(M)
1	20	293.15	1	66.7	3.33
	20	293.15	3	66.7	3.33
2	10	283.15	1	74.1	2.59
	10	283.15	3	74.1	2.59
3	0	273.15	1	79.4	2.06
	0	273.15	3	79.4	2.06
4	-10	263.15	1	85.5	1.45
	-10	263.15	3	85.5	1.45

[a] Conditions: [M]:cat. = 100:1, [C4GBL] = 10 M in THF (0.15 g, 1.34 mmol), [b] Determined by ^{1}H NMR.

To investigate the thermodynamic parameters of C4GBL, experiments were carried out at an initial monomer concentration of $[M]_0$ = 10 M. Typically, a flame-dried Schlenk tube was charged with ^{t}BuP$_4$ (16.7 µL) and THF (134 µL) in a glove box. The

tube was then removed from the glove box and immersed in a cooling bath maintained at the desired temperature (20, 10, 0, or –10 °C). After equilibration at the target temperature for 10 min under stirring, C4GBL (150 mg, 1.34 mmol) was injected to initiate polymerization. At predetermined time intervals (1 h and 3 h), small aliquots of the reaction mixture were withdrawn and quenched with a benzoic acid solution in $CDCl_3$. The monomer conversion of C4GBL was determined by ^{1}H NMR spectroscopy. The polymerization was considered to have reached equilibrium when the monomer conversion remained constant over two consecutive measurements.

$$\mathrm{Ln}([\mathrm{M}]_{\mathrm{eq}})\ \grave{}= \Delta H_{\mathrm{p}}/R\mathrm{T} - \Delta S_{\mathrm{p}}/R \qquad (1)$$

where T is the temperature at which polymerization takes place and *R* is the gas constant. The slope and intercept give access to the values of ΔH and ΔS, respectively

Calculated ΔG for different temperatures are based on equation 2

$$\Delta G = \Delta H - \mathrm{T}\Delta S \qquad (2)$$

Calculated Tc for different concentrations are based on equation 3

$$T_{\mathrm{c}} = \Delta H_{\mathrm{p}}/(\Delta S_{\mathrm{p}} + R\ln[\mathrm{M}]_0) \qquad (3)$$

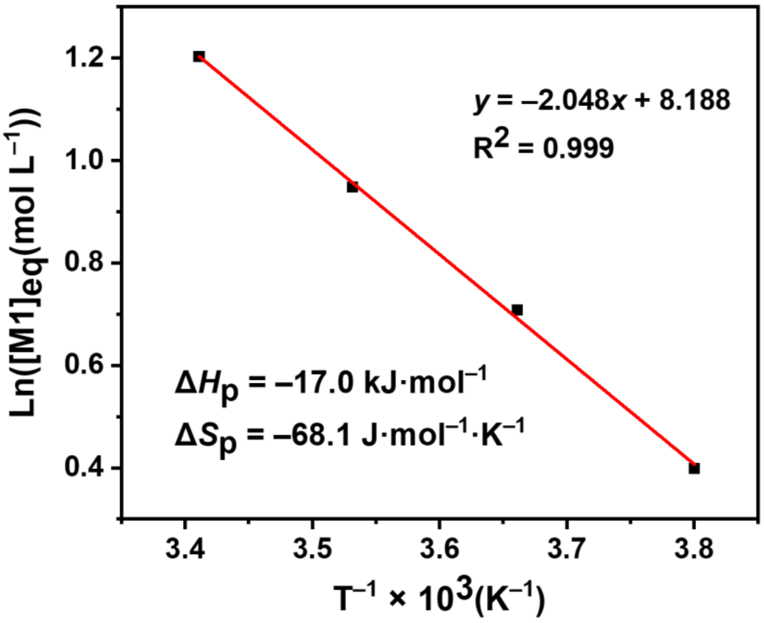

Figure S19. Van't Hoff plot of the ^{t}BuP$_4$-catalyzed ROP of C4GBL ([C4GBL]/[^{t}BuP$_4$] = 100/1, $[M]_0$ = 10 mol L^{-1} in THF).

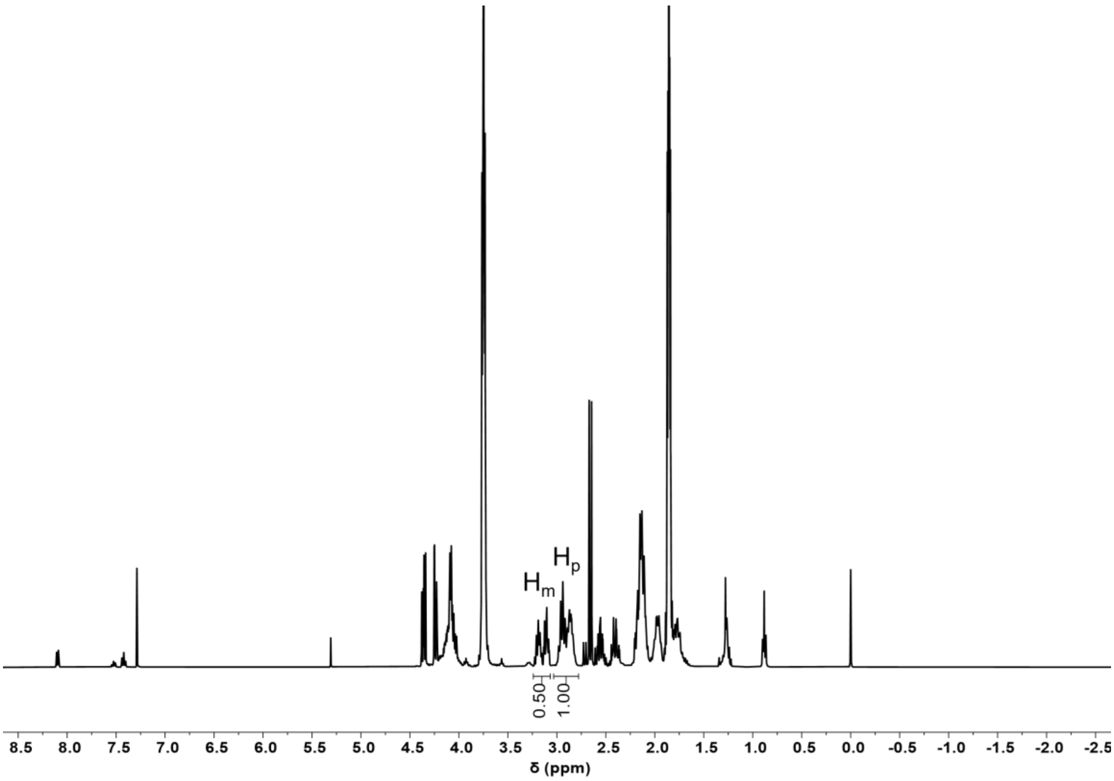

Figure S20. Crude ¹H NMR spectrum used to calculate the equilibrium conversion at 20 ℃ after 1 h.

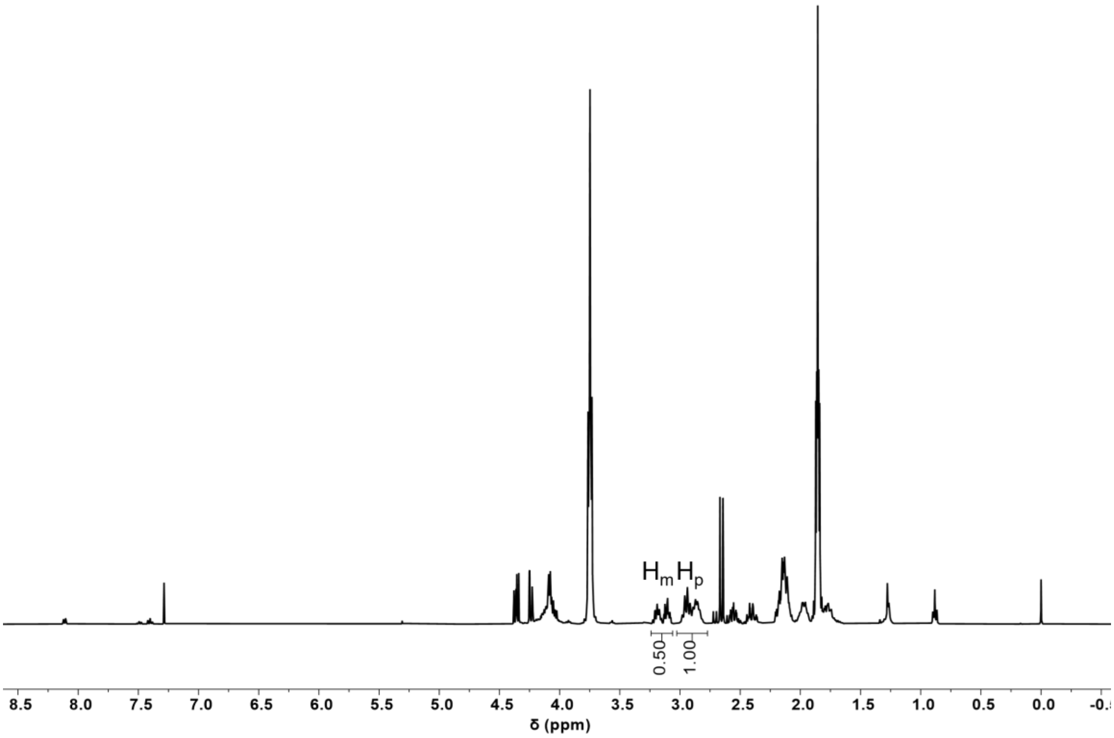

Figure S21. Crude ¹H NMR spectrum used to calculate the equilibrium conversion at 20 ℃ after 3 h.

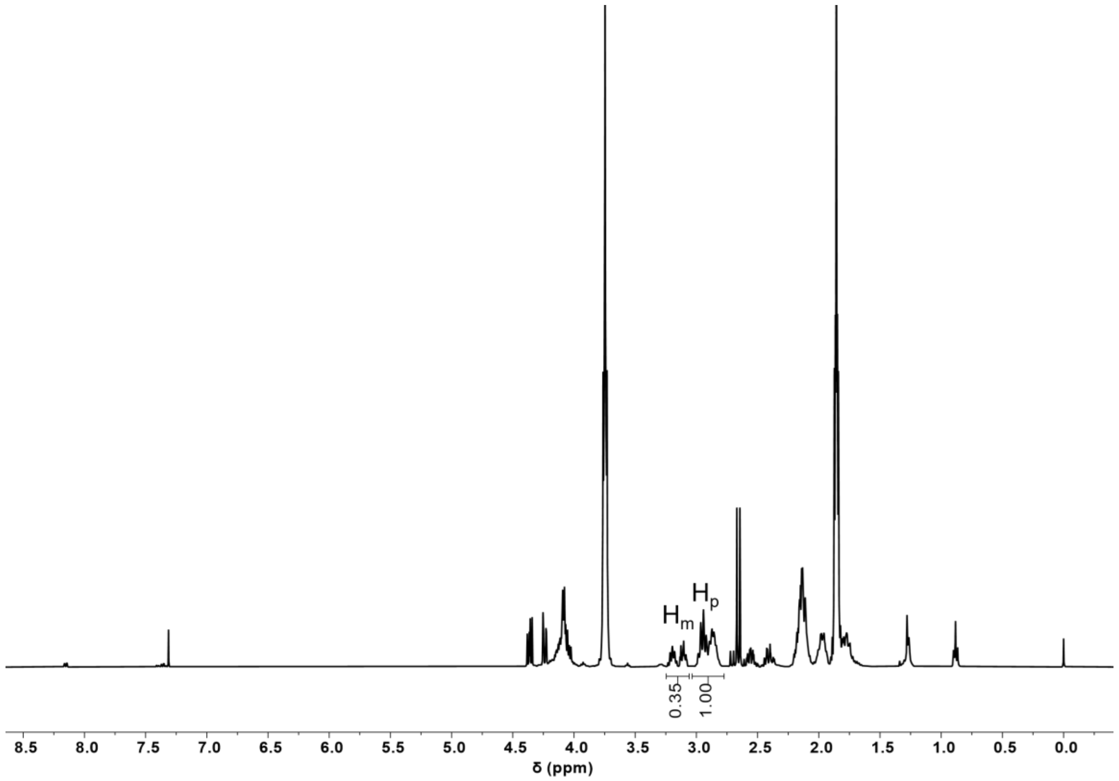

Figure S22. Crude ^{1}H NMR spectrum used to calculate the equilibrium conversion at 10 ℃ after 1 h.

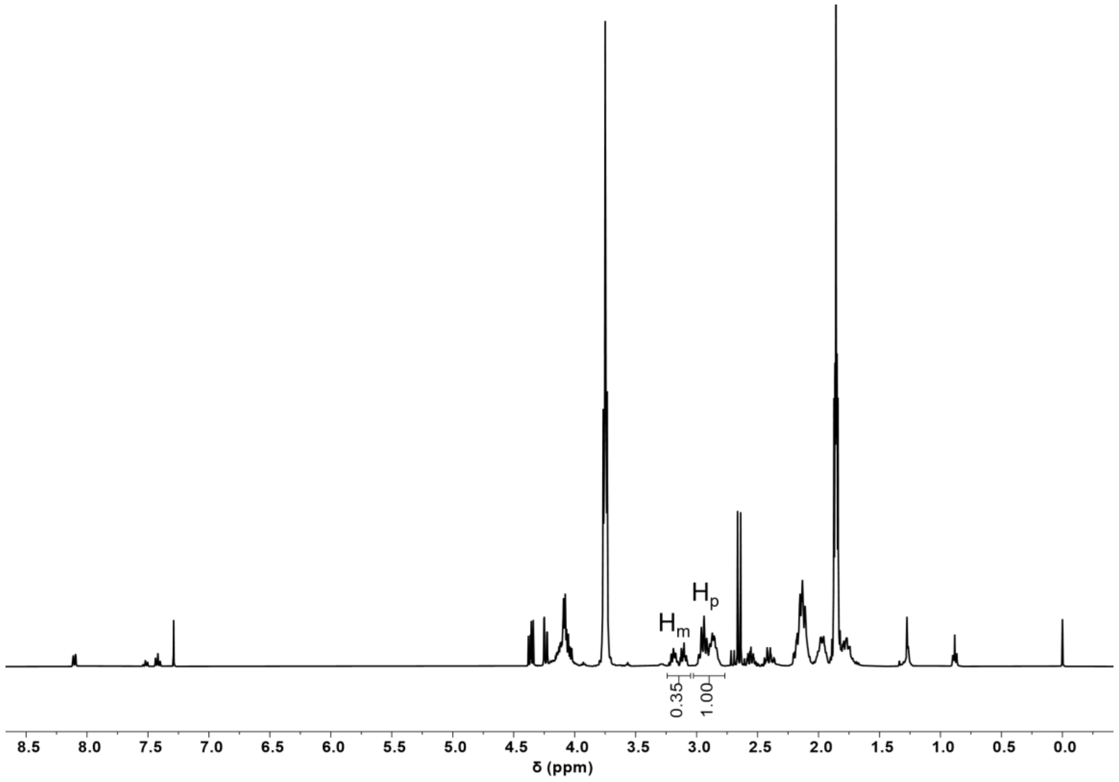

Figure S23. Crude ^{1}H NMR spectrum used to calculate the equilibrium conversion at 10 ℃ after 3 h.

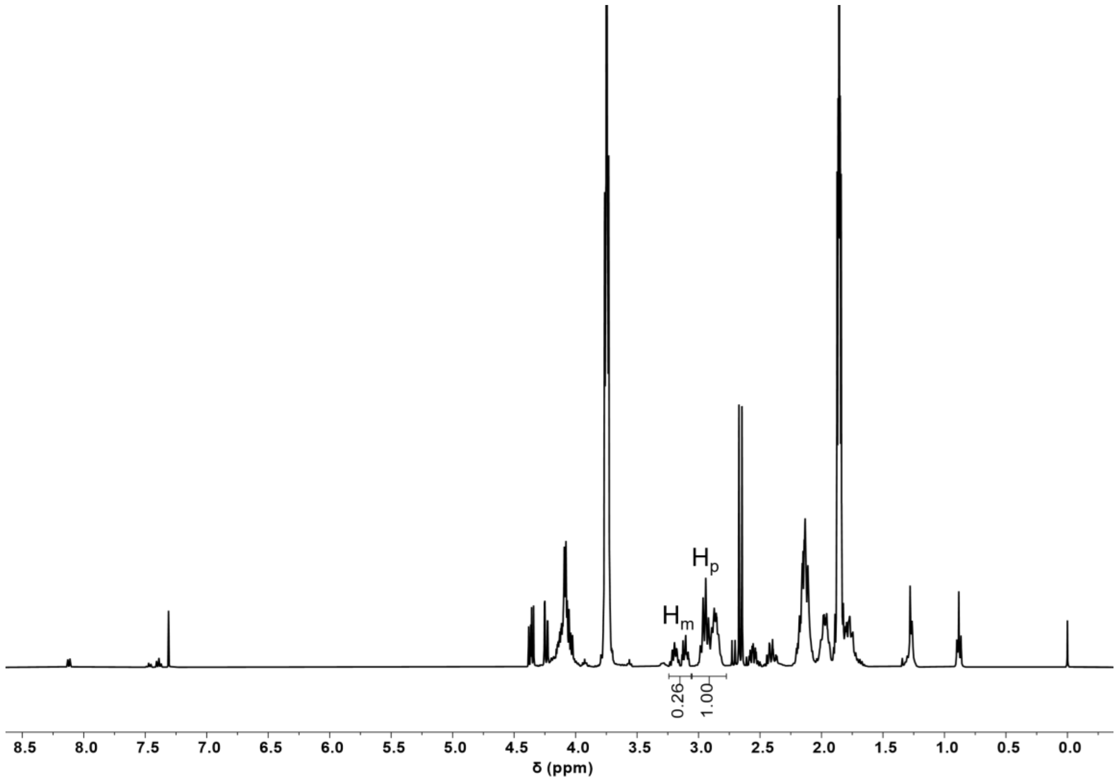

Figure S24. Crude ^{1}H NMR spectrum used to calculate the equilibrium conversion at 0 ℃ after 1 h.

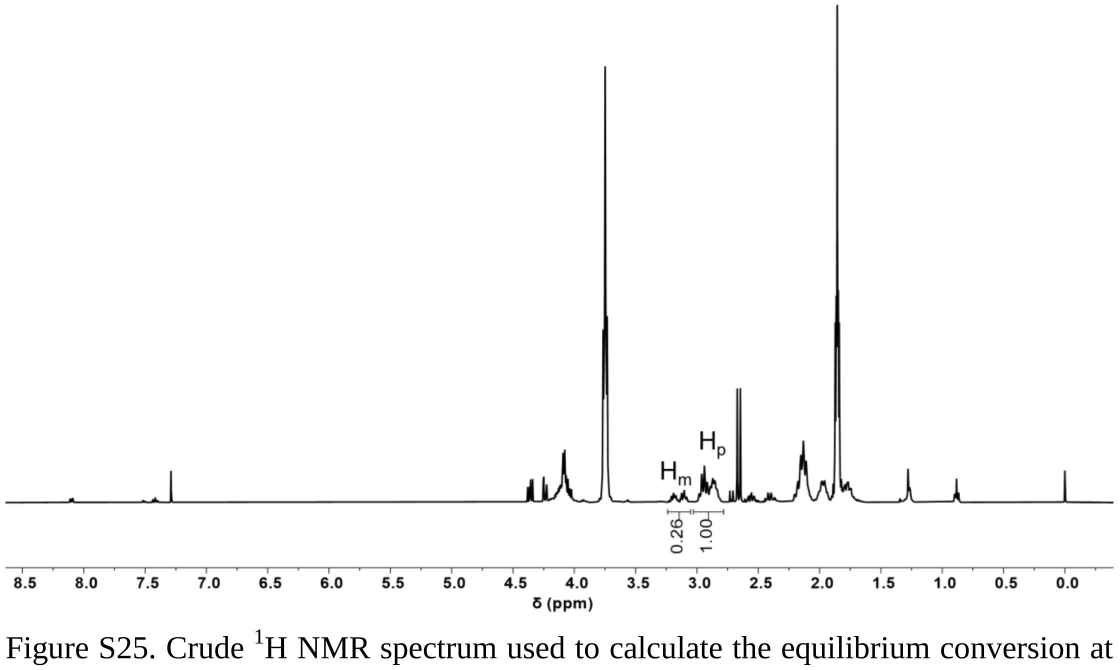

Figure S25. Crude ^{1}H NMR spectrum used to calculate the equilibrium conversion at 0 ℃ after 3 h.

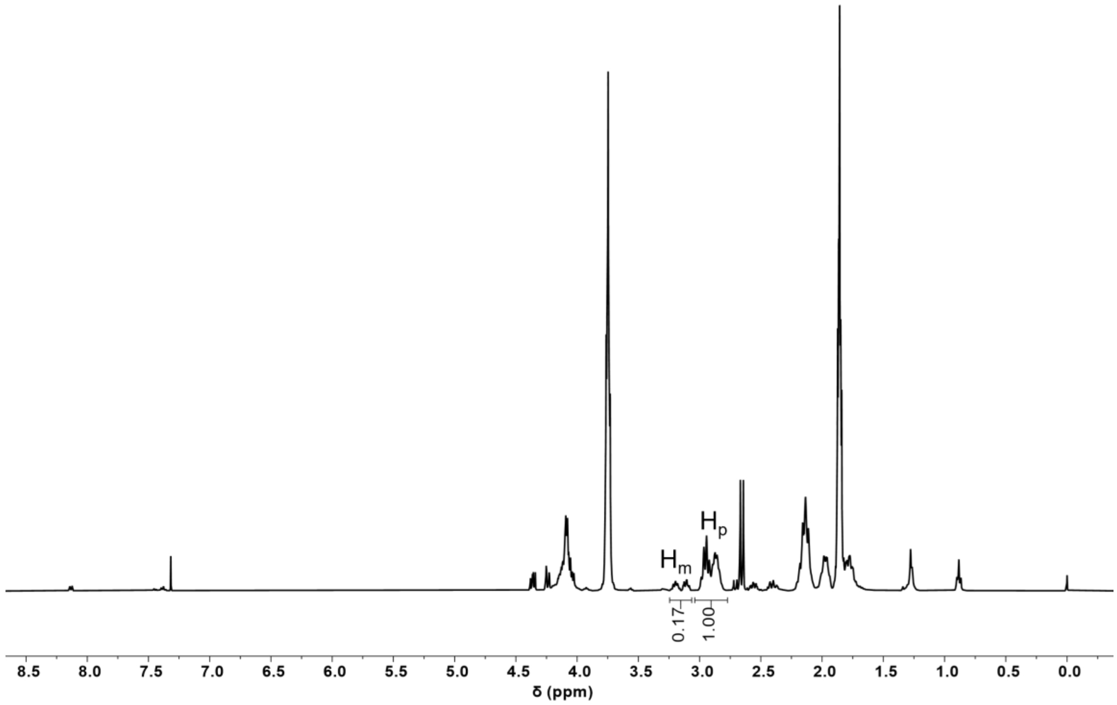

Figure S26. Crude ^{1}H NMR spectrum used to calculate the equilibrium conversion at −10 ℃ after 1 h.

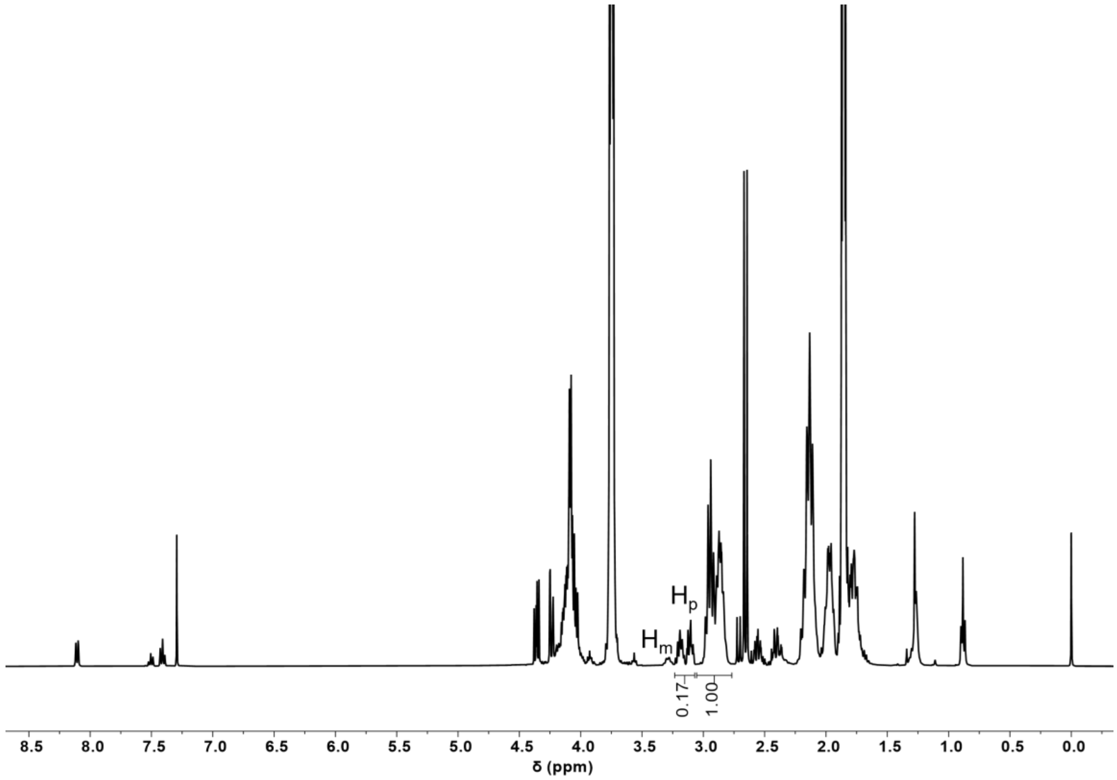

Figure S27. Crude ^{1}H NMR spectrum used to calculate the equilibrium conversion at −10 ℃ after 3 h.

Chemical recycling of poly(C4GBL).

Depolymerization catalyzed by ^{t}BuOK.

P(C4GBL) (1.4 g, 12.5 mmol of repeating units) and ^{t}BuOK (70 mg, 5 mol% relative to the repeating unit) were placed in a flame-dried single-neck round-bottom flask equipped with a magnetic stir bar. The reaction mixture was then immersed in a preheated oil bath and stirred at 150 °C for 4 h. During this period, gradual depolymerization of the polyester occurred, and volatile products were collected by distillation under reduced pressure. The regenerated monomer was obtained as a colorless liquid after purification by silica gel column chromatography.

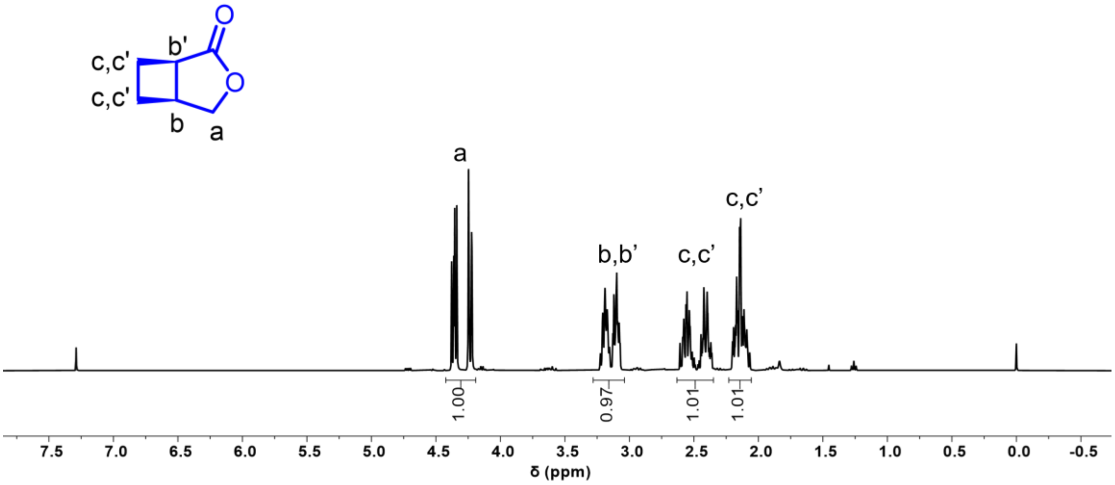

Figure S28. ^{1}H NMR spectrum in $CDCl_3$ of the unpurified monomer obtained by depolymerization of the polymer using ^{t}BuOK.

Depolymerization catalyzed by tin(II) 2-ethylhexanoate ($Sn(Oct)_2$).

P(C4GBL) (0.80 g, 7.14 mmol of repeating units) and $Sn(Oct)_2$ (145 mg, 5 mol% relative to the repeating unit) were placed in a flame-dried single-neck round-bottom flask equipped with a magnetic stir bar. The reaction mixture was immersed in a preheated oil bath and stirred at 150 °C. Only a small amount of C4GBL was recovered by vacuum distillation during the reaction. After 4 h, the reaction mixture was cooled to room temperature, dissolved in dichloromethane, and precipitated into

cold methanol. The resulting solid was collected and dried under vacuum at 50 °C for 12 h. The dried material was subsequently analyzed by NMR and GPC.

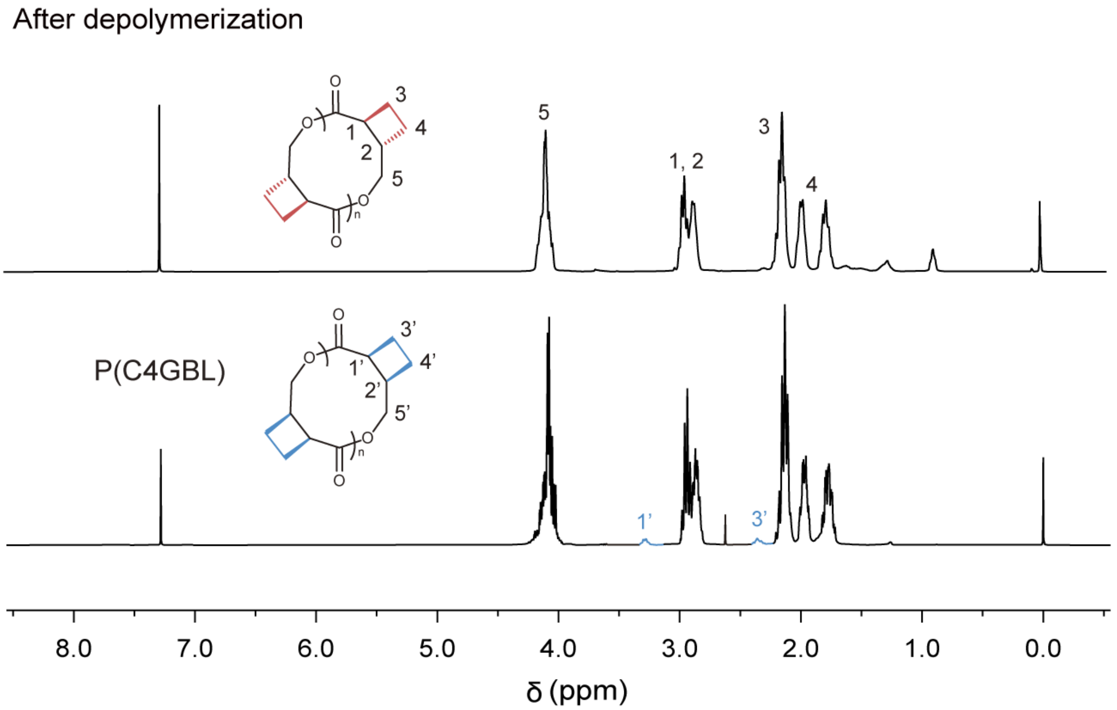

Figure S29. Overlay of the ^{1}H NMR spectra in $CDCl_3$ of poly(C4GBL) (bottom) and the $Sn(Oct)_2$-catalyzed depolymerization residue (top).

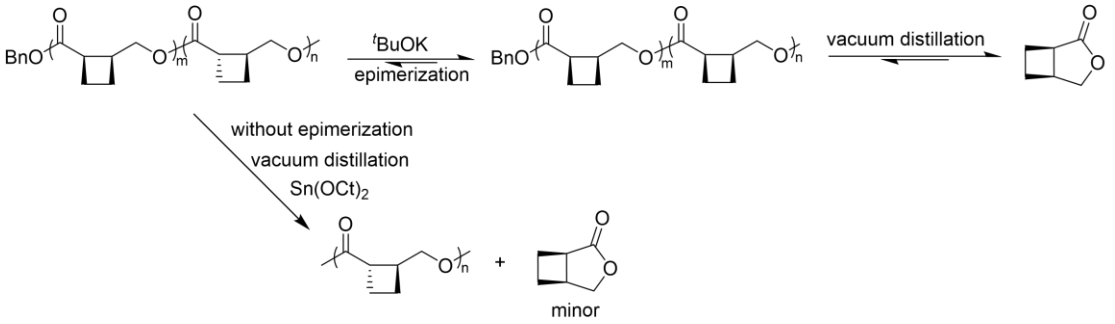

Scheme S3. Proposed depolymerization pathways under various catalysts.

Repolymerization of recovered monomer.

Recovered C4GBL monomer was purified by column chromatography and thoroughly dried prior to use. In a glovebox, ^{t}BuP$_4$ (16.7 μL, [M]:[cat.] = 200:1) was dissolved in THF (268 μL) in a dry Schlenk tube, and recovered C4GBL (0.30 g, 2.68 mmol) was added. The mixture was stirred at 17 ℃ for 1 h. The polymer was precipitated into cold methanol (40mL), collected by filtration, and dried under vacuum at 50 ℃ for 12 h.

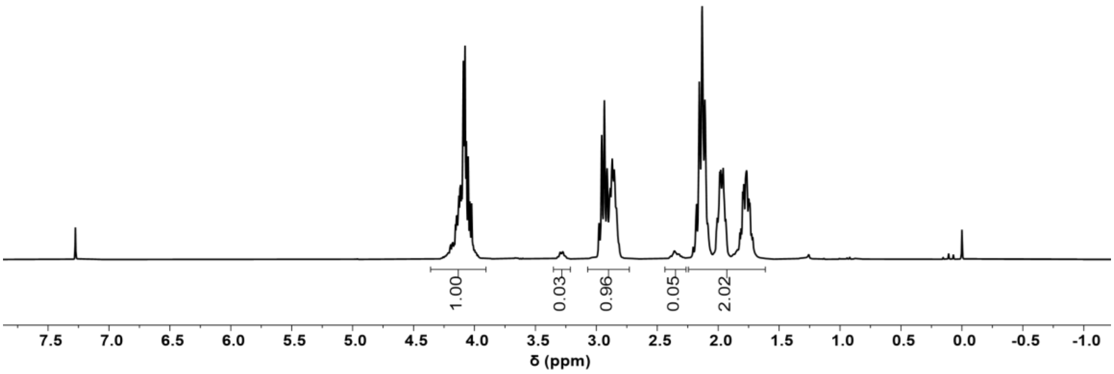

Figure S30. ^1H NMR (400 MHz, $CDCl_3$ 25 ℃) spectrum of P(C4GBL) obtained from repolymerization of recovered C4GBL.

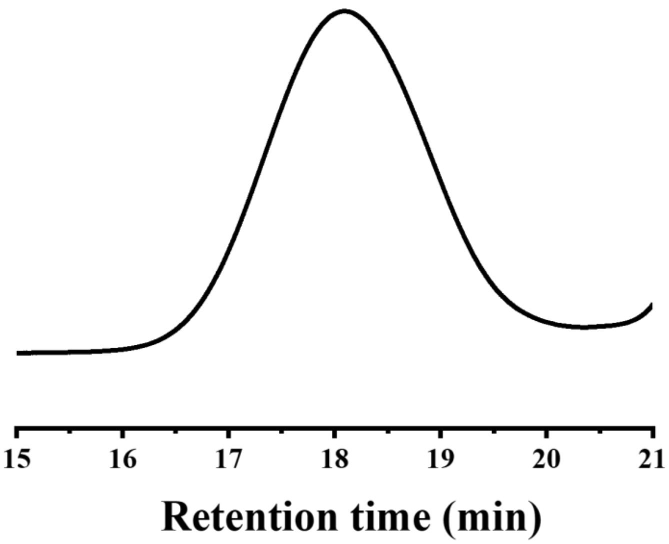

Figure S31. SEC trace of the linear polymer prepared using the ^{t}Bu-P_4/Ph_2CHOH system (Entry 1, Table 1).

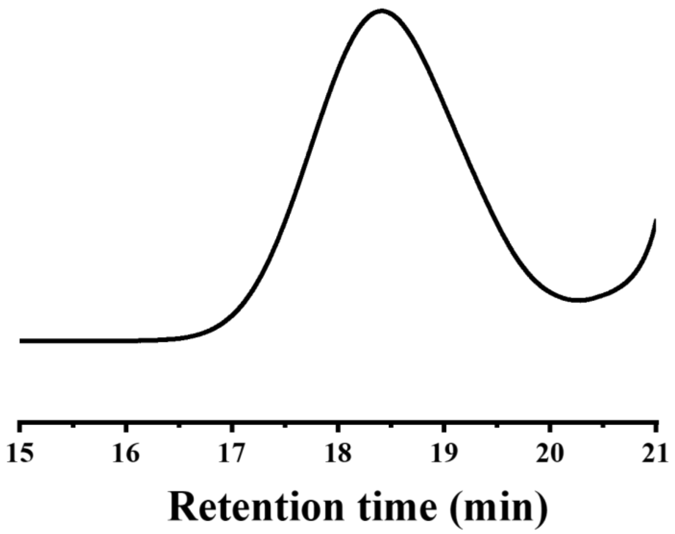

Figure S32. SEC trace of the linear polymer prepared using the ^{t}Bu-P_4/BnOH system (Entry 5, Table 1).

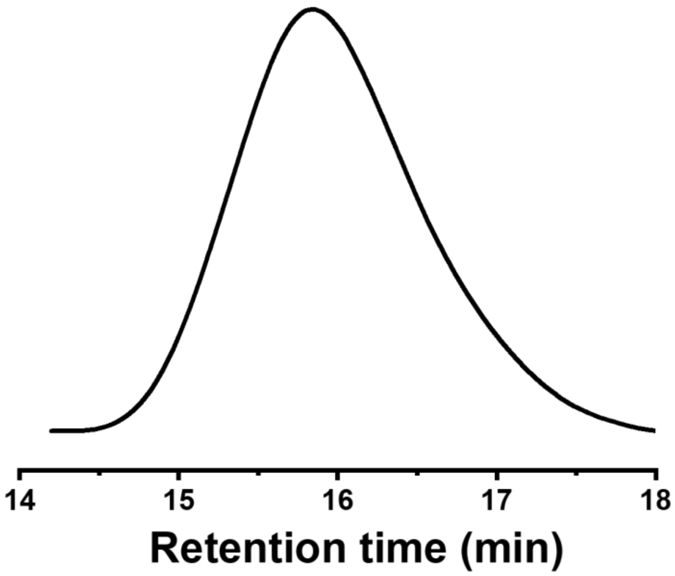

Figure S33. SEC trace of the cyclic polymer prepared using ^{t}BuP_4 alone (Entry 7, Table 1).

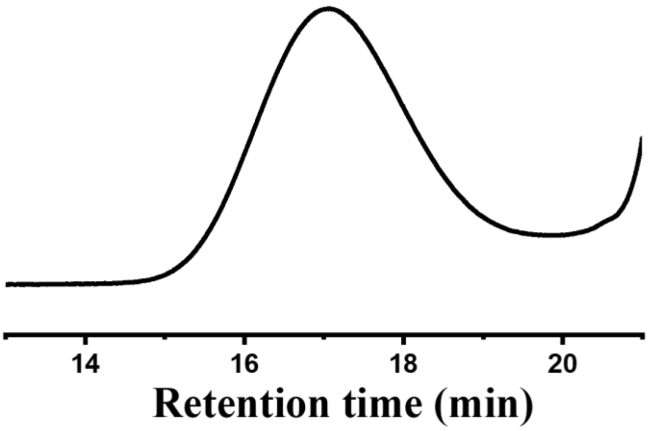

Figure S34. SEC trace of the cyclic polymer prepared using $^{t}BuP_4$ alone (Entry 13, Table 1).

Computational details

Density functional theory (DFT) calculations were performed using the B3LYP functional with all electron 6-311 + G(d,p) basis set on all atoms as implemented in Gaussian 16 C.01 program.[2] Grimme's D3 correction scheme with Becke-Johnson damping was used in all calculations.[3] Nature of the stationary points was confirmed by the vibrational analysis carried out at the same level of theory. All structures corresponding to local minima showed no imaginary frequencies. Reaction Gibbs free energies (ΔG_{298K}) were computed using the results of the normal-mode analysis within the ideal gas approximation at a pressure of 1 atm and temperature of 298.15 K. Coordinates of optimized structures are presented in the log files, which can be found in the data repository, as well as the input files.

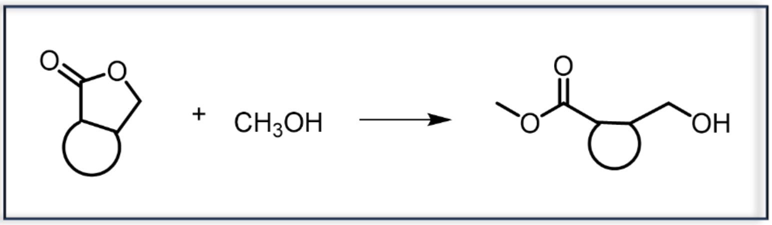

Table S3. Computed energies of lactone ring-opening with methanol.

structure	Energies lactone monomers		Energies methanol adducts		Energy of ring opening reaction		Experimental T_c (℃)
	H (a.u.)	G (a.u.)	H (a.u.)	G (a.u.)	ΔH (kcal mol^{-1})	ΔG (kcal mol^{-1})	
**MeOH**	-115.712701	-115.739778	n.a.	n.a.	n.a.	n.a.	n.a.
**3,4-S6GBL**	-462.507121	-462.550099	-578.225748	-578.277375	-3.719	7.845	-
**GBL**	-306.496152	-306.530871	-422.220991	-422.267518	-7.617	1.965	-136
**C4GBL**	-383.874979	-383.913218	-499.652996	-499.650221	-9.165	1.741	-24
**4,5-T6GBL**	-462.513878	-462.555822	-578.242649	-578.295055	-10.084	0.342	4
**3,4-T6GBL**	-462.510246	-462.552223	-578.240656	-578.292508	-11.113	-0.318	0

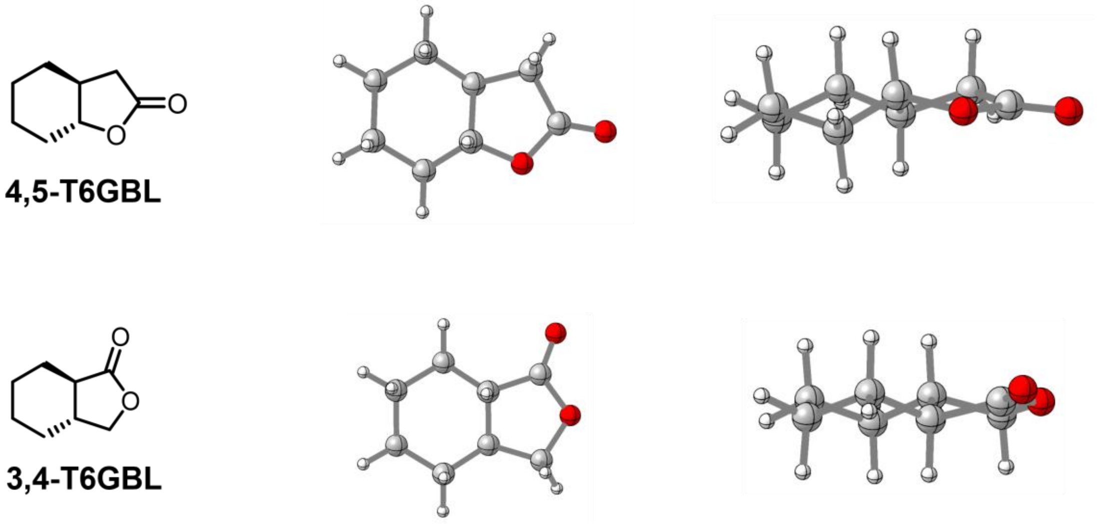

Figure S35. Optimized twist conformations of lactones 4,5-T6GBL and 3,4-T6GBL, obtained from DFT calculations.

Optimized geometries from DFT calculations at M062x/6-31g(d,p) level.

3,4-S6GBL

C	0.6245	0.73678	0.32626
C	0.57662	–0.6551	–0.29727
C	1.97469	–1.14436	0.0375
O	2.78796	0.00111	–0.07656
C	2.04251	1.14362	–0.00516
O	2.45609	2.27124	–0.15444

H	0.58971	0.67564	1.4419
H	0.08435	–1.3548	0.42502
H	2.3327	–1.93032	–0.66421
H	2.04019	–1.52599	1.08206
C	–0.76571	1.36089	0.41585
C	–0.48898	–0.7654	–1.3093
C	–1.85208	–0.63023	–0.65699
C	–1.82814	0.29108	0.54829
H	–1.62776	–0.30916	1.4749
H	–0.96772	1.96405	–0.50858
H	–0.37381	0.01892	–2.10321
H	–0.41015	–1.76355	–1.81299
H	–2.21954	–1.64019	–0.33639
H	–2.58172	–0.24196	–1.41507
H	–2.83305	0.77155	0.6736
H	–0.82481	2.06096	1.28932

GBL

C	–0.7656	0.1633	–0.1917
C	–0.4488	–1.2838	0.1791
C	1.0402	–1.3607	–0.1249
O	1.5482	–0.0622	0.039
C	0.5555	0.867	0.0362
O	0.7101	2.0597	0.1779
H	–1.0277	0.2577	–1.2713
H	–1.5796	0.5983	0.4306
H	–0.6201	–1.4405	1.2713
H	–1.054	–2.0263	–0.3883
H	1.2197	–1.6714	–1.1807
H	1.5796	–2.0597	0.5533

C4GBL

C	–0.73591	0.06384	–0.10599
C	–0.41966	–1.36764	0.32157
C	1.07194	–1.45285	0.03288
O	1.5763	–0.14806	0.15197

C	0.58203	0.77833	0.10608
O	0.73337	1.97583	0.20408
H	–0.98925	0.11704	–1.19049
H	–1.01885	–2.13219	–0.22243
H	1.26069	–1.80266	–1.00898
H	1.60694	–2.12474	0.74129
C	–1.66346	–1.32634	1.20638
C	–1.95016	0.12013	0.75145
H	–2.43973	–2.05911	0.90216
H	–1.46323	–1.42818	2.2934
H	–2.89463	0.23609	0.17977
H	–1.91891	0.86763	1.57146

4,5-T6GBL

C	0.0464	0.663	–0.2967
C	0.0202	–0.741	0.2838
C	1.4139	–1.2081	–0.1183
C	2.1938	0.0877	0.0616
O	1.3277	1.1423	–0.0277
C	–1.0936	1.4823	0.292
C	–2.4165	0.7909	–0.0974
C	–2.4556	–0.700	0.296
C	–1.2233	–1.4826	–0.2012
O	3.3861	0.1757	0.2534
H	–0.0634	0.6354	–1.4093
H	–0.0076	–0.6881	1.402
H	1.4584	–1.5193	–1.1868
H	1.7986	–2.0243	0.5329
H	–1.0658	2.5244	–0.1049
H	–1.0008	1.5417	1.4014
H	–2.5543	0.8695	–1.2028
H	–3.2742	1.3297	0.3718
H	–3.3861	–1.1734	–0.099
H	–2.5062	–0.7722	1.4093
H	–1.2289	–1.5445	1.3142
H	–1.2289	–2.5244	0.1961

3,4-T6GBL

C	0.5932	0.7094	0.2976
C	0.6002	−0.6957	−0.2976
C	2.0095	−1.1303	0.0644
O	2.7851	0.0395	−0.064
C	2.0008	1.1572	−0.0254
O	2.378	2.2949	−0.1931
C	−0.612	1.4877	−0.2278
C	−1.8869	0.7338	0.2001
C	−1.8791	−0.7472	−0.2288
C	−0.6016	−1.4879	0.2136
H	0.5465	0.6695	1.4138
H	0.5413	−0.6308	−1.4138
H	2.4027	−1.9182	−0.616
H	2.0743	−1.4877	1.1176
H	−0.5654	1.5683	−1.3388
H	−0.6138	2.5237	0.1855
H	−2.7851	1.2457	−0.2208
H	−1.9804	0.7818	1.3117
H	−1.9616	−0.7969	−1.3412
H	−2.7767	−1.267	0.1838
H	−0.582	−2.5237	−0.199
H	−0.5686	−1.5654	1.3252

References

[1] J. Seo, S. Y. Lee, C. W. Bielawski, *Macromolecules* **2020**, *53*, 3202−3208.

[2] Frisch, M. J. et al. G16_C01. Gaussian 16, Revision C.01, Gaussian, Inc., Wallin at (2016).

[3] S. Grimme, J. Antony, S. Ehrlich, H. A Krieg, *J. Chem. Phys.* **2010**, *132*, 154101.
